# Correlations between sleep problems, core symptoms, and behavioral problems in children and adolescents with autism spectrum disorder: a systematic review and meta-analysis

**DOI:** 10.1007/s00787-023-02253-1

**Published:** 2023-07-21

**Authors:** Heeyeon Kim, Jae Han Kim, Ju Hyeon Yi, Jong Yeob Kim, Marco Solmi, Samuele Cortese, Lee Smith, Ai Koyanagi, Jae Il Shin, Keun-Ah Cheon, Paolo Fusar-Poli

**Affiliations:** 1 Department of Psychiatry, Yongin Severance Hospital, Yonsei University College of Medicine, Yongin, Republic of Korea; 2 Institute of Behavioral Science in Medicine, Yonsei University College of Medicine, Yonsei University Health System, Seoul, Republic of Korea; 3 Yonsei University College of Medicine, Severance Hospital, Yonsei University Health System, Seoul, Republic of Korea; 4 Department of Psychiatry, University of Ottawa, Ottawa, ON Canada; 5 Department of Mental Health, The Ottawa Hospital, Ottawa, ON Canada; 6 Clinical Epidemiology Program, Ottawa Hospital Research Institute, University of Ottawa, Ottawa, ON Canada; 7 Department of Child and Adolescent Psychiatry, Charité Universitätsmedizin, Berlin, Germany; 8 Centre for Innovation in Mental Health, School of Psychology, Faculty of Environmental and Life Sciences, University of Southampton, Southampton, UK; 9 Clinical and Experimental Sciences (CNS and Psychiatry), Faculty of Medicine, University of Southampton, Southampton, UK; 10 Solent NHS Trust, Southampton, UK; 11 Hassenfeld Children’s Hospital at NYU Langone, New York University Child Study Center, NY New York City, USA; 12 Centre for Health, Performance, and Wellbeing, Anglia Ruskin University, Cambridge, CB1 1PT UK; 13 Parc Sanitari Sant Joan de Déu/CIBERSAM/ISCIII, Sant Boi de Llobregat, Barcelona Spain; 14 ICREA, Pg. Lluis Companys 23, Barcelona, Spain; 15 Department of Pediatrics, Yonsei University College of Medicine, Yonsei-ro 50, Seodaemun-gu, C.P.O. Box 8044, Seoul, 03722 Republic of Korea; 16 Severance Children’s Hospital, Yonsei University Health System, Seoul, Republic of Korea; 17 Severance Underwood Meta-research Center, Institute of Convergence Science, Yonsei University, Seoul, Republic of Korea; 18 Department of Child and Adolescent Psychiatry, Yonsei University College of Medicine, Severance Hospital, Yonsei-ro 50, Seodaemun-gu, Seoul, 03722 Republic of Korea; 19 Department of Psychosis Studies, Early Psychosis: Interventions and Clinical-Detection (EPIC) Lab, Institute of Psychiatry, Psychology and Neuroscience, King’s College London, London, UK; 20 Department of Brain and Behavioral Sciences, University of Pavia, Pavia, Italy; 21 OASIS Service, South London and Maudsley NHS Foundation Trust, London, UK; 22 National Institute for Health Research, Maudsley Biomedical Research Centre, London, UK

**Keywords:** Autism spectrum disorder, Sleep problems, Core symptoms, Behavioral problems, Correlation, Meta-analysis

## Abstract

Children and adolescents with autism spectrum disorder (ASD) experience various sleep problems. Sleep problems co-occur in a bidirectional relationship with ASD core symptoms and behavioral problems. However, studies on how these three factors are intricately linked to each other are limited. This meta-analysis examined the differential relationship between specific sleep problems, core symptoms, and behavioral problems in this population. This study was registered in PROSPERO (CRD42022339695). We systematically searched the PubMed/MEDLINE, Web of Science, and Scopus databases from inception to April 27, 2022. Observational studies that reported correlations between measures of sleep problems, ASD core symptoms, or ASD behavioral problems were included, and participants aged 18 years or below were enrolled. The correlation coefficient (*r*) was assessed as the primary effect metric. Total 22 cross-sectional studies were included, which comprised 2655 participants (mean age = 6.60 years old; mean percentage of boys = 80.64%). We found correlations between total sleep problems and total core symptoms (*r* 0.293 [95% confidence interval − 0.095 to 0.604]), total sleep problems and total behavioral problems (*r* 0.429 [0.299–0.544]), and total core symptoms and total behavioral problems (*r* − 0.050 [− 0.177 to 0.079]) and identified statistically significant correlations between specific components of sleep problems, ASD core symptoms, and ASD behavioral problems. Each specific sleep problem showed a unique association with core symptoms and behavioral problems. Sleep problems in ASD should be explored in detail, and the closely linked core symptoms and behavioral problems should be common therapeutic targets.

**Supplementary Information:**

The online version contains supplementary material available at 10.1007/s00787-023-02253-1.

## Introduction

Autism spectrum disorder (ASD) is characterized by two core symptoms (social communication problems and restricted and repetitive behavior) and usually accompanies various profiles of behavioral problems [1, 2]. Children and adolescents with ASD often experience sleep problems, with a prevalence ranging from 40 to 80% [3]. Compared to typically developing individuals whose sleep problems improve with aging, sleep problems in these individuals arise as early as toddlerhood and persist and vary during adolescence or even throughout life [4, 5]. Many reports have shown that sleep problems are associated with ASD core symptoms or behavioral problems [6, 7]. Previous studies have found that the use of melatonin for sleep problems in individuals with ASD can concomitantly improve social communication [8], stereotyped behaviors [9], and behavior and daytime functioning [10]. In addition, considering that sleep problems may increase parental stress and negatively affect the family's quality of life, monitoring and appropriate intervention for sleep problems may be needed from the beginning of treatment.

Since the causes of sleep problems have not been specifically identified, several models have been proposed to explain the inter-relatedness between sleep problems and other psychopathologies. According to the biopsychosocial model [3], a combination of environmental stimuli, intrinsic physiological abnormalities, and behavioral characteristics related to core symptoms contribute to sleep problems. On the other hand, the bidirectional theoretical framework [11] suggests a bidirectional relationship in which core symptom severity acts as a vulnerability factor for sleep problems; conversely, sleep problems exacerbate core symptoms through behavioral problems. While there is significant phenotypic overlap and interaction between core symptoms and behavioral problems in ASD, the core symptoms are traits that persist as development progresses, whereas concurrent behavioral problems, potentially induced by environmental or psychological factors, can improve when external influences are regulated.

The status of our knowledge concerning the relationships among sleep problems, core symptoms, and behavioral problems remains dynamically interrelated; however, relatively little is known regarding how each specific sleep problem is differentially related. Specific sleep problems experienced by individuals with ASD can encompass shortened sleep time, prolonged sleep latency, lower sleep efficiency [12, 13], bedtime resistance, parasomnia, and decreased percentage of rapid eye movement (REM) sleep [14–16]. Furthermore, it is unclear whether there is a combination of significantly related specific sleep problems, types of autistic symptoms, and patterns of behavioral problems. Investigation into this combination could phenotype sleep problems, providing information on psychopathology that may not have been discovered and establishing a common therapeutic target. Using a meta-analytic approach, this study aimed to examine the differential relationship between specific sleep problems, core symptoms, and behavioral problems in children and adolescents with ASD.

## Methods

The present systematic review and meta-analysis were prepared following the Preferred Reporting Items for Systematic Reviews and Meta-analyses guidelines (Appendix pp. 4–7) [17]. The protocol was registered in PROSPERO (CRD42022339695).

### Search strategy and selection criteria

**Fig. 1:**
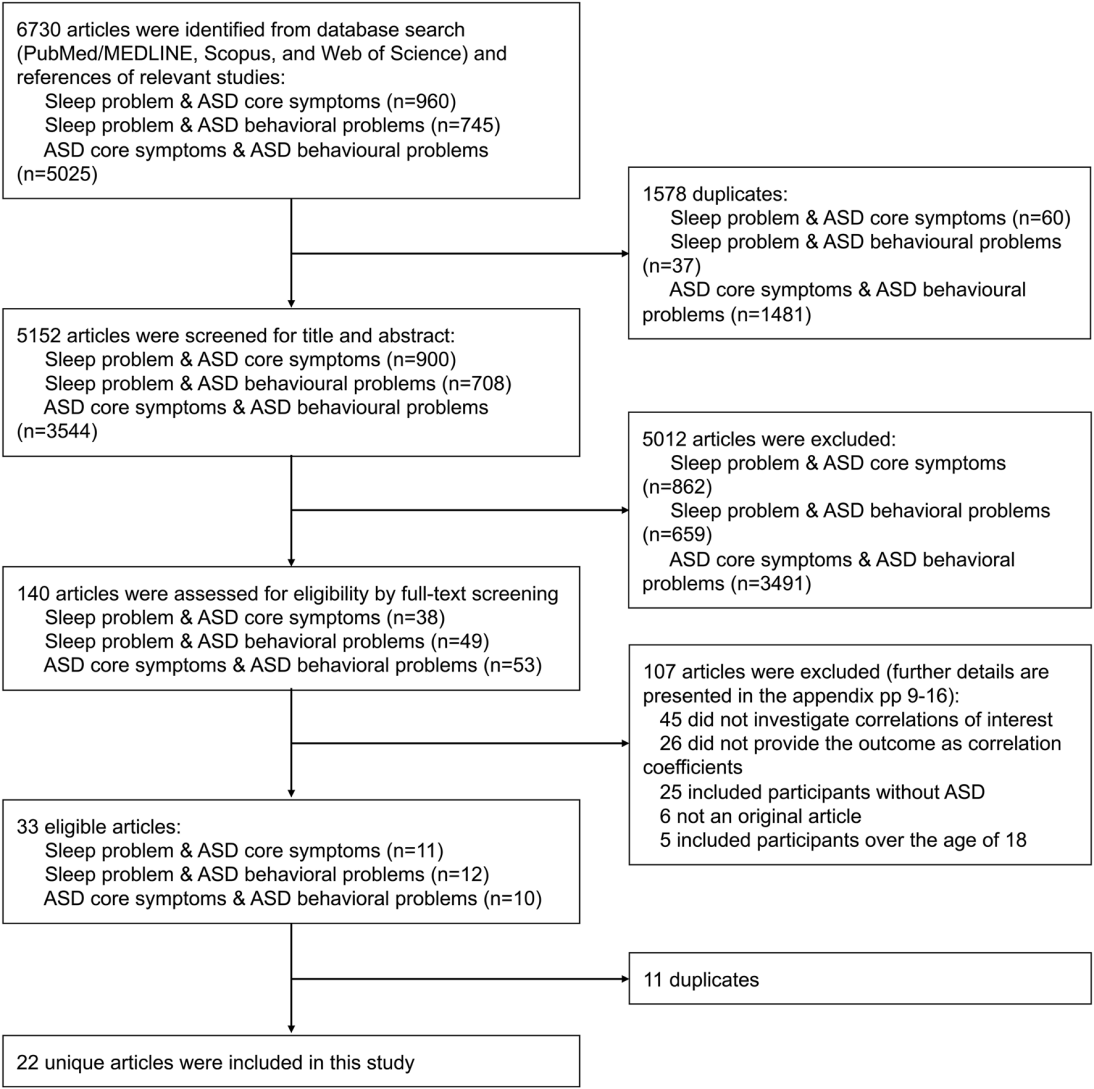
Flow diagram for study selection. *ASD* autism spectrum disorder

We systematically searched the PubMed/MEDLINE, Web of Science, and Scopus databases from inception to April 27, 2022. No language restrictions were imposed. Since we investigated three-way correlations between sleep problems, ASD core symptoms, and ASD behavioral problems, we performed systematic searches for each correlation, that is, three times in total (Fig. 1). The full search terms for each database are available on Appendix p. 8. Two independent authors (JHK and JHY) screened titles, abstracts, and full texts sequentially (Fig. 1), and reference lists of the relevant studies were also observed to identify further eligible studies. Any disagreements were resolved through a consensus with other authors (HK, JIS, or KAC).

We included observational studies that reported correlation coefficients (*r*) between measures of either (i) sleep problems, (ii) ASD core symptoms, or (iii) ASD behavioral problems and enrolled ASD participants aged 18 years or below. The clarification of sleep problems, ASD core symptoms, and ASD behavioral problems is described below. ASD diagnosis was operationalized according to any version of the Diagnostic and Statistical Manual of Mental Disorders (DSM), International Classification of Diseases, Autism Diagnostic Observation Schedule (ADOS), Autism Diagnostic Interview (ADI), Social Responsiveness Scale (SRS), or Social Communication Questionnaire (SCQ) on the basis of the clinician’s interview. We excluded a study that included participants without ASD, with participants over 18 years, with no investigations on the abovementioned correlations of interest, and the outcome was not provided as correlation coefficients. Additionally, a paper that is not an original article was also excluded.

### Data extraction

Two independent authors (JHK and JHY) extracted the following data from eligible studies: name of the first author, publication year, country of study, diagnostic methods used to define ASD, details of the study (number of participants, mean age and standard deviation, percentage of boys, mean intelligence quotient [IQ], medication use status [yes or no]), and outcome of the study (correlation coefficient, name of the measurement tool for sleep problems, behavioral problems, or core symptoms of ASD). Any disagreements were resolved by a discussion with another author (HK).

### Study quality assessment

The quality of the included studies was evaluated by two independent authors (JHK and JHY) using the Appraisal Tool for Cross-Sectional Studies (AXIS) because all included articles had a cross-sectional design [18]. The AXIS tool contains 20 questions that are categorized into introduction (one question), methods (10 questions), results (five questions), discussion (two questions), and others (two questions). Each question can be answered by “yes,” or “no,” or “do not know,” and the total score ranges from 0 to 20.

### Data analysis

We performed a meta-analysis of correlation coefficients (*r*) between specific components of (i) sleep problems (bedtime resistance, daytime sleepiness, night waking, parasomnias, sleep anxiety, sleep duration, sleep-disordered breathing, sleep-onset delay, and total sleep problems), (ii) ASD core symptoms (social communication problems, restricted and repetitive behavior, and total ASD core symptoms), and (iii) ASD behavioral problems (affective/anxiety problems, somatic complaints, anxious/depressive problems, thought problems, attention problems, aggressive/delinquent problems, internalizing problems, externalizing problems, and total behavioral problems). Clarification of sleep problems, ASD core symptoms, and ASD behavioral problems and its details are presented in Appendix pp 17–20. Analysis was performed without distinguishing adjusted and unadjusted estimates since related information was not provided by all the included studies. The following process was automatically conducted by R package “metafor” (version 3.0–2), which includes the “metacor” function: (1) Fisher’s z transformation that converts each correlation coefficient into a z-value, (2) combination of the results, and (3) inverse Fisher’s z transformation that calculates correlation coefficients and corresponding 95% confidence interval (95% CI) from the pooled z-values. Considering the expected heterogeneity, random-effects models were used. According to Cohen’s convention, the effect sizes of the correlation were deemed to be very small (*r* < 0.1), small (0.1 ≤ *r* < 0.3), moderate (0.3 ≤ *r* < 0.5), and large (*r* ≥ 0.5).

For evaluating between-study heterogeneity, we conducted Cochran’s Q test, which produces Q statistics (the magnitude of statistical heterogeneity) and estimated *I*^2^ statistics (the proportion of variance in the pooled effect size attributable to the heterogeneity) [19]. To assess publication bias, we visually inspected funnel plots and performed Egger’s test [20]. When Egger’s test suspected publication bias, we used the trim-and-fill method to adjust for publication bias by correcting the effect size [21].

Meta-regression was performed for continuous variables (mean age of participants, percentage of boys, mean IQ of participants, and AXIS score) and subgroup analysis for a categorical variable (medication use status [yes or no]). R software (version 4.1.3) and RStudio (version 1.4.1717) were used for statistical tests. All statistical tests were two-sided, and statistical significance was set at *P* < 0.05.

## Results

### Study selection and study characteristics

From systematic searches for each correlation of (i) sleep problems and ASD core symptoms, (ii) sleep problems and ASD behavioral problems, and (iii) ASD core symptoms and ASD behavioral problems, we identified 5152 candidate articles after removing duplicates, of which 11, 12, and 10 articles, respectively, were found to be eligible after the screening process (Fig. 1). Notably, 22 unique articles were included because of duplication. The list of excluded studies in the full-text screening phase for each search is provided in Appendix pp. 9–16.

The characteristics of each included study are presented in Table 1. A total of 2655 participants (median 84 per study, interquartile range 57.75–170, range 14–437) were included. The mean age of participants was 6.60 years, the mean percentage of boys was 80.64%, and the mean IQ was 85.86. Three of the 22 studies (14%) included medication-naïve patients with ASD.

**Table 1:** Characteristics of included studies

Author (year)	Country	Diagnostic tool/criteria	*N*	Percentage of boys (%)	Mean age ± SD (years)	Mean IQ	Medication use	AXIS score	Measurement for sleep problems	Measurement for behavioral problems	Measurement for ASD core symptoms
Cremone-Caira (2019)	USA	ADOS, ADI-R	101	86.14	9.13 ± 1.38	106.12	Yes	17	CSHQ	CBCL	
Factor (2017)	USA	DSM-5	57	82.46	7.25 ± 3.85	90.98	Yes	20		CBCL	SRS-2
Fadini (2015)	Brazil	DSM-5	45	78	9.7 ± 4.1	NR	Yes	18	SDSC	CBCL	
Gabriels (2005)	USA	DSM-IV, ADOS	14	71.42	10.58 ± 6.98	81.43	Yes	18	CSQ	Aberrant Behavior Checklist	RBS-R
Galligan (2021)	USA	ADOS-2	233	80.7	5.1 ± 1.0	84.8	Yes	18		CBCL	SRS, ADOS
Gunes (2019)	Turkey	DSM-5	112	72.3	8.06 ± 3.22	NR	No	20	CSHQ		CARS
Hirata (2016)	Japan	DSM-5, ADOS-G	193	80.83	4.45 ± 1.24	77.28	Yes	20	JSQ-P	CBCL	
Kang (2020)	China	DSM-5	252	80.6	5.13 ± 1.15	NR	No	20	CSHQ	SDQ	RBQ-2, SDQ, CARS
Kim (2021)	Republic of Korea	DSM-IV, ADOS, ADI-R	96	94.79	14.3 ± 1.80	98.91	Yes	20		CBCL	SRS-2
Manelis-Baram (2022)	Israel	DSM-5, ADOS-2	83	NR	NR	NR	Yes	20	CSHQ		ADOS-2
Mazurek (2016)	USA	ADOS or ADOS-2	81	86.4	10.3 ± 3.8	NR	Yes	20	CSHQ	C-SHARP, VADPRS	
Mazurek (2019)	USA	DSM-IV, ADOS	437	82.6	5.07 ± 2.14	76.24	Yes	20	CSHQ	CBCL	Aberrant Behavior Checklist
McVey (2018)	USA	ADOS-G	113	87.6	13.47 ± 1.41	103.81	Yes	20		CBCL	SRS
Muskett (2019)	USA	DSM-5, ADOS-2, ADI-R	35	82.9	7.6 ± 2.98	93.83	Yes	20		CBCL	RBS-R
Mutluer (2016)	Istanbul	DSM-IV-TR	64	79.69	11.66 ± 3.8	NR	No	20	PSQ	CBCL	Aberrant Behavior Checklist
Phung (2018)	USA	SCQ, ADOS-2	28	89.3	14.64 ± 1.97	95.5	Yes	20	Sleep Habit Survey	CBCL	
Reynolds (2017)	USA	DSM-IV-TR, ADOS, ADI-R	85	75.29	9.29 ± 1.79	NR	Yes	19	CBCL	CPRS, CBCL	ADI-R
Saito (2017)	Japan	SRS	189	57.7	5.39 ± 0.49	NR	Yes	20		SDQ	SRS
Thenhausen (2017)	Germany	ICD-10	15	86.67	14.32 ± 3.03	NR	Yes	18	SDSC	CBCL	CBCL, SRS
Wang (2016)	China	DSM-IV-TR	60	83.3	11.53 ± 2.92	NR	Yes	20	CSHQ	SDQ	SDQ
Wang (2019)	China	DSM-IV or DSM-5	81	82.72	5.18 ± 0.92	NR	Yes	20	CSHQ	SDQ	Short sensory profile
Zaidman-Zait (2020)	Canada	DSM-IV-TR, ADOS, ADI-R	281	84.7	4.41 ± 0.35	NR	Yes	20	CSHQ	CBCL	ADOS

*ADI* Autism Diagnostic Interview, *ADI*-R Autism Diagnostic Interview-Revised, *ADOS* Autism Diagnostic Observation Schedule, *ADOS*-G Autism Diagnostic Observation Schedule—Generic, *ASD* autism spectrum disorder, *AXIS* the Appraisal tool for Cross-Sectional Studies, *C-SHARP* the Children’s Scale for Hostility and Aggression, *CARS* Childhood Autism Rating Scale, *CBCL* child behavior checklist, *CPRS* Conners Parent Rating Scale, *CSHQ* The Children’s Sleep Habits Questionnaire, *CSQ* Child Sleep Questionnaire, *DSM* Diagnostic and Statistical Manual of Mental Disorders, *ICD* International Classification of Diseases, *IQ* intelligence quotient, *JSQ-P* Japanese Sleep Questionnaire for Preschoolers, *N* the number of patients, *NR* not reported, *PSQ* Pediatric Sleep Questionnaire, *RBQ* Repetitive Behaviors Questionnaire, *RBS-R* Repetitive Behavior Scales-Revised, *SD* standard deviation, *SDQ* Strengths and Difficulties Questionnaire, *SDSC* Sleep Disturbance Scale for Children, *SRS* Social Responsiveness Scale, *VADPRS* Vanderbit ADHD Diagnostic Parent Rating Scale

### Correlations between specific sleep problems and ASD core symptoms

**Fig. 2:**
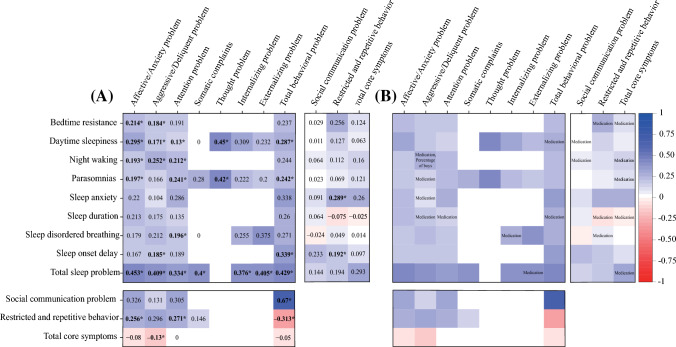
Results of **A** meta-analysis and **B** meta-regression and subgroup analysis. Note: **A** Statistically significant correlations are highlighted with bold and symbol (*), and **B** only statistically significant results are displayed

Correlations between sleep problems and ASD core symptoms were reported in 11 studies (references are listed in Appendix p. 21). Among the identified studies, correlations between sleep anxiety and restricted and repetitive behavior (number of participants [*N*] = 252, *r* = 0.289 [95% CI 0.172–0.398]), and sleep-onset delay and restricted and repetitive behavior (*N* = 252, *r* = 0.192 [0.070–0.308]) were statistically significant, while others were not (Fig. 2A, Appendix pp. 26–28). Detailed information on publication bias detection and effect size correction are displayed in Appendix p. 29.

### Correlations between specific sleep problems and ASD behavioral problems

Correlations between sleep problems and behavioral problems were reported in 12 studies (references are listed in Appendix p. 22). Among the identified correlations, the following 24 symptom pairs were positively correlated with statistical significance: correlations between total sleep problems and affective/anxiety problem (*N* = 828, *r* = 0.453 [95% CI 0.336–0.557]), daytime sleepiness and thought problems (*N* = 45, *r* = 0.450 [0.180–0.657]), total sleep problems and total behavioral problem (*N* = 584, *r* = 0.429 [0.299–0.544]), parasomnias and thought problems (*N* = 45, *r* = 0.420 [0.144–0.635]), total sleep problems and aggressive/delinquent problem (*N* = 828, *r* = 0.409 [0.211–0.575]), total sleep problems and externalizing problems (*N* = 272, *r* = 0.405 [0.009–0.691]), total sleep problems and somatic complaints (*N* = 452, *r* = 0.400 [0.269–0.517]), total sleep problems and internalizing problems (*N* = 357, *r* = 0.376 [0.197–0.531]), sleep-onset delay and total behavioral problem (*N* = 312, *r* = 0.339 [0.022–0.594]), total sleep problems and attention problem (*N* = 1014, *r* = 0.334 [0.261–0.403]), daytime sleepiness and affective/anxiety problem (*N* = 636, *r* = 0.295 [0.218–0.369]), daytime sleepiness and total behavioral problem (*N* = 565, *r* = 0.287 [0.119–0.439]), night waking and aggressive/delinquent problem (*N* = 674, *r* = 0.252 [0.060–0.427]), parasomnias and total behavioral problems (*N* = 520, *r* = 0.242 [0.135–0.343]), parasomnias and attention problem (*N* = 493, *r* = 0.241 [0.185–0.296]), bedtime resistance and affective/anxiety problem (*N* = 593, *r* = 0.214 [0.107–0.316]), night waking and attention problem (*N* = 674, *r* = 0.212 [0.055–0.359]), parasomnias and affective/anxiety problem (*N* = 327, *r* = 0.197 [0.062–0.325]), sleep-disordered breathing and attention problem (*N* = 753, *r* = 0.196 [0.107–0.282]), night waking and affective/anxiety problem (*N* = 593, *r* = 0.193 [0.014–0.360]), sleep-onset delay and aggressive/delinquent problem (*N* = 393, *r* = 0.185 [0.002–0.356]), bedtime resistance and aggressive/delinquent problem (*N* = 674, *r* = 0.184 [0.114–0.252]), daytime sleepiness and aggressive/delinquent problem (*N* = 689, *r* = 0.171 [0.047–0.289]), and daytime sleepiness and attention problem (*N* = 774, *r* = 0.130 [0.053–0.206]) (Fig. 2A, Appendix pp. 30–33). Detailed information on publication bias detection and effect size correction are displayed in Appendix p. 34.

### Correlations between ASD core symptoms and ASD behavioral problems

Correlations between behavioral problems and core symptoms of ASD were reported in 10 studies (references are listed in Appendix p. 23). Among the identified correlations, the following three symptom pairs were positively correlated with statistical significance: correlations between total behavioral problems and social communication problem  (*N* = 233, *r* = 0.670 [95% CI 0.593–0.735]), affective/anxiety problem and restricted and repetitive behavior (*N* = 1222, *r* = 0.256 [0.058–0.434]), and attention problem and restricted and repetitive behavior (*N* = 1077, *r* = 0.271 [0.055–0.463]). Negative correlations were detected for correlations between total behavioral problems and restricted and repetitive behavior (*N*= 81, *r* = − 0.313 [− 0.497 to − 0.102]) and between aggressive/delinquent problems and total core symptoms (*N* = 281, *r* = − 0.130 [− 0.243 to − 0.013]) (Fig. 2A, Appendix pp. 35–36). Detailed information on publication bias detection and effect size correction are displayed in Appendix p. 37.

### Meta-regression and subgroup analyses

Meta-regression and subgroup analyses found that some estimates were moderated by medication use status or percentage of boys but not by other potential moderators (mean age of participants and AXIS score). Meta-regression for the mean IQ of participants was unavailable because of the small number of reported studies (*k* < 4).

**Fig. 3:**
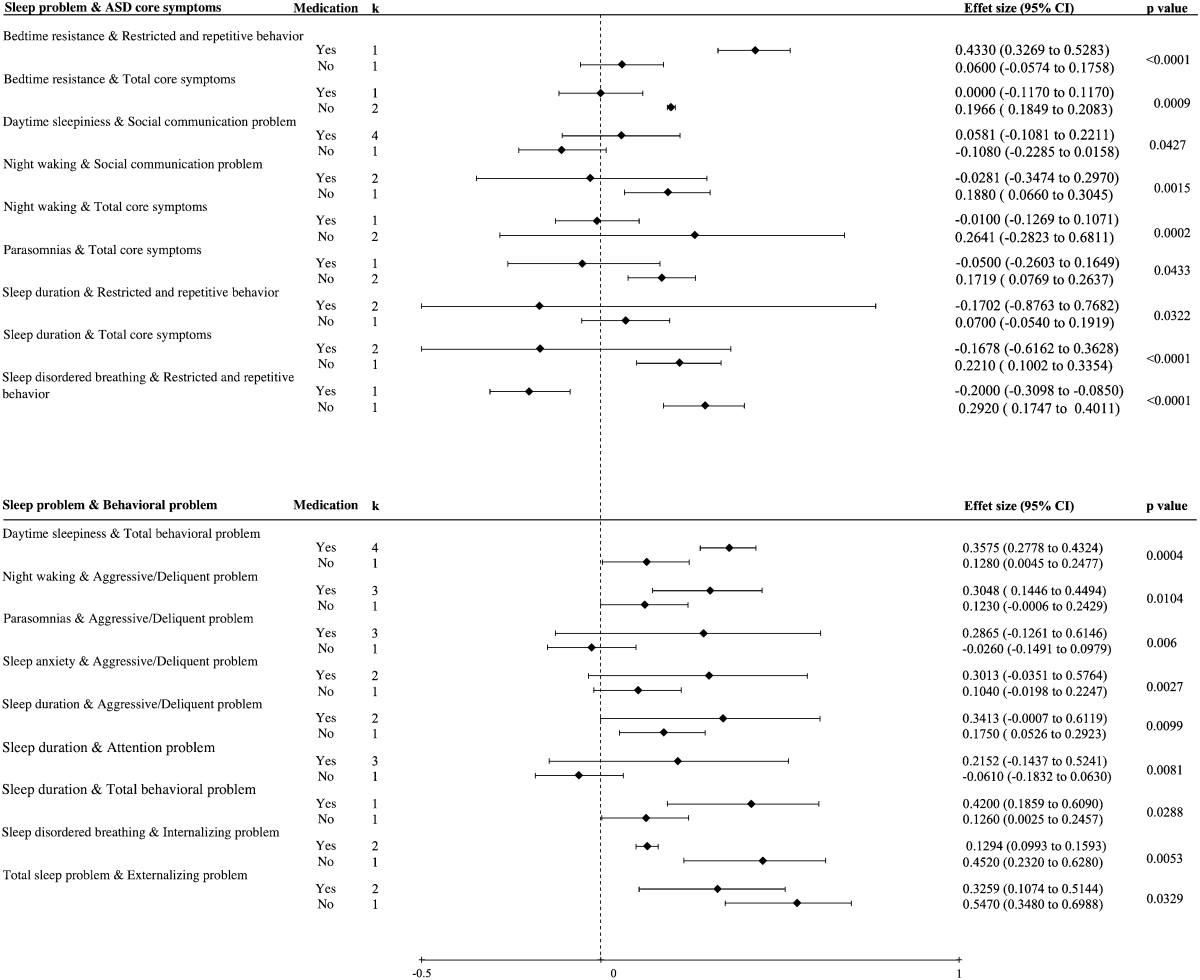
Detailed results of subgroup analysis of medication use status. *ASD* autism spectrum disorder, *CI* confidence interval, *k* the number of studies

Our meta-regression found that the correlation between night waking and aggressive/delinquent problems was higher when the percentage of boys was higher (regression coefficient 0.0468 [95% CI 0.0269–0.0668], *P* = 0.0096), while other correlations were not (Fig. 2B). Subgroup analysis identified numerous estimates that were moderated by medication use status (Fig. 2B). Notably, the magnitude of the correlation in the medication-use group tended to be smaller than that in the medication-naive group for correlations between sleep problems and ASD core symptoms while it was reversed for correlations between sleep problems and ASD behavioral problems (Fig. 3). Subgroup analysis of medication use status for correlations between behavioral problems and ASD core symptoms was not possible because all related studies included only participants under medication use. Detailed statistics for meta-regression and subgroup analyses are presented in Appendix pp. 38–50.

### Study quality assessment

The total AXIS scores for each included study are shown in Table 1, where 16 of 22 (73%) studies received a maximum score of 20 points. Of note, the other six studies were deducted mainly because of unsatisfactory study methods. Details of AXIS scoring for each study with reasons are provided in Appendix pp. 24–25.

## Discussion

To the best of our knowledge, this is the first systematic review and meta-analysis to comprehensively explore the interrelatedness between specific sleep problems, ASD core symptoms, and behavioral problems among children and adolescents with ASD. Our findings from 22 studies, including a total of 2655 participants, may offer a more precise understanding of the link between sleep and other psychopathologies.

In the relationship between specific sleep problems and ASD core symptoms, restricted and repetitive behaviors were significantly correlated with sleep anxiety and sleep-onset delay, whereas social communication problems did not show a significant correlation with any specific sleep problems. However, most ASD behavioral problems were significantly correlated with several parameters of sleep problems, among which affective/anxiety problems showed the highest correlation with total sleep problems. When medication-naive patients were excluded, the relationships between specific sleep problems and ASD behavioral problems became more pronounced, especially in aggressive/delinquent problems. Finally, in terms of the correlations between ASD core symptoms and ASD behavioral problems, restricted and repetitive behaviors were associated with both affective/anxiety and attention problems. Age (mean age of participants), sex (percentage of boys), and cognitive ability (mean IQ of participants) did not significantly moderate any of the identified correlations, except for sex, which moderated the correlation between night waking and aggressive/delinquent behavior.

Daytime sleepiness is a sleep problem commonly observed in clinical practice, and its prevalence in children with ASD was reported to be 14.7% [22]. In addition, unlike the normal development population whose sleep problems usually improve with aging, daytime sleepiness in ASD patients seemed to be intensified in adolescence [23], which may call for continuous follow-up. Reports of slow melatonin metabolism in ASD, which leads to altered circadian rhythm and elevated melatonin levels in the daytime are not only biological grounds for daytime sleepiness but also suggest a connection with the pathophysiology of ASD [24, 25]. In this study, daytime sleepiness was not significantly associated with ASD core symptoms, while it is moderately correlated with affective/anxiety symptoms, which may suggest that, as implied in the previous study [26], excessive sleepiness in the daytime can be accompanied by affective/anxiety symptoms regardless of the severity of autistic symptoms. Excessive sleepiness during the day can lead to a vicious cycle of fewer learning opportunities and social interactions with friends, which is essential for these individuals, resulting in greater isolation and a depressed mood.

Bedtime resistance, sleep anxiety, and sleep-onset delay indicate (i) resisting and struggling to sleep in their own bed simultaneously, (ii) having to be next to someone because of the fear of darkness or being alone, and (iii) having difficulty falling asleep in 20 min even after lying down [27], all of which refer to a delay in the sleeping process. In this meta-analysis, bedtime resistance was significantly associated with affective/anxiety and aggressive/delinquent behavior. When the medication-naïve group was excluded, bedtime resistance was moderately correlated with restricted and repetitive behaviors. Considering that our analysis found that both sleep anxiety and sleep-onset delay were significantly correlated with restricted and repetitive behavior, it can be inferred that all three types of sleep problems were closely associated with restricted and repetitive behavior. Previous studies have documented that the severity of restricted and repetitive behavior is associated with a higher prevalence of sleep problems in children with ASD [28]. Moreover, a study that involved preschool-aged ASD patients showed that bedtime resistance explained 20.6% of the variance in the restricted and repetitive behavior severity score through linear regression analysis [29]. Several studies using the revised version of ADI suggested that restricted and repetitive behavior can be reliably parsed into two subtypes, repetitive sensory-motor and insistence on sameness [30, 31]. Repetitive sensory-motor includes repetitive use of objects, complex motor mannerisms, and sensory-seeking behavior [31, 32], whereas rituals, compulsions, and resistance to change in routines are examples of insistence on sameness [33]. In one study that examined specific sleep problems in ASD found that sleep-onset delay and bedtime resistance were related to repetitive sensory-motor, but no correlation was found with insistence on sameness [34]. The results of this meta-analysis can be interpreted in this context, and it can be inferred that among sleep problems, the difficulty in settling and initiating sleep is deeply related to atypical sensory experience. Approximately 60–90% of patients with ASD are reported to have sensory features, including problems with sensory reactivity, perception, and integration [35, 36]. With an unexpected sensory experience, it is more difficult to disengage from the sensory environment, and hyper-reactivity leads to atypical behavioral responses and an elevation of physiological arousal, making it difficult to fall asleep. Against this background, it can be expected that for children with a low sensory threshold, concurrent sensory integration therapy will improve sleep problems and ASD core symptoms.

Night waking and parasomnia are other sleep problems that children with ASD struggle with [37], and these sleep problems showed the strongest correlations with behavioral problems in some previous studies [6, 38]. Night waking in ASD differs from typically developing children in that they wake up and may simply laugh, vocalize, or play with an object for 2–3 h [3, 28, 39, 40]. In this study, night waking was moderately correlated with aggressive/delinquent behavior, which appeared to be greater in males. Children with ASD tend to experience difficulties in emotional regulation, and negative valence and high arousal affect their sleep architecture. By reducing slow-wave sleep or disrupting REM sleep [41], emotional dysregulation may cause individuals to wake up during sleep. In contrast, parasomnia, including night terror and walking, was significantly related to attentional problems, and this relationship has been reported in children with attention-deficit/hyperactivity disorder (ADHD), as these sleep problems were associated with inattention severity and improved with methylphenidate [42, 43]. Problems in sleep duration did not seem to be significantly correlated with ASD core symptoms or behavioral problems, but short sleep duration was moderately associated with some problematic behavior in a subgroup analysis, except for the medication-naïve group. If medication-naive patients had milder overall autistic symptoms, sleep duration might be associated with behavioral problems in patients with higher levels of ASD.

As expected, previous studies have reported that the severity of ASD core symptoms is positively related to various types of problematic behaviors [44, 45]. If it is possible to identify specific sleep problems commonly associated with ASD core symptoms and behavioral problems that are closely related to each other, a multilateral analysis of the factors affecting sleep will be possible. In this study, restricted and repetitive behavior had significant relationships with affective/anxiety and attention problems, and social communication problems showed a high correlation with overall behavioral problems; however, there were no closely related specific behavioral problems. Consequently, a combination of sleep problems, ASD core symptoms, and behavioral problems that are closely related to each other could not be identified. This may be because the meta-analysis of the relationship between core symptoms and behavioral problems showed a relatively high heterogeneity. When excluding medication-naive groups, bedtime resistance, restricted and repetitive behavior, and affective/anxiety problems were closely associated with each other, suggesting that close examination of the other two factors is needed to help ASD patients who struggle in bed.

Some limitations should be addressed. First, the small number of included studies in each meta-analysis lowered the statistical power and led to an incomplete subgroup analysis and meta-regression, which should be carefully considered when interpreting our results. Second, we included similar parameters of different questionnaires in the meta-analysis (e.g., “nightmares” of CBCL, “parasomnias” of CSHQ, and “disorders of arousal” of SDSC), which may partly contribute to the large heterogeneity of some results. Since all sleep problems were measured using a subjective questionnaire, objective sleep alteration through polysomnography or actigraphy should be supplemented in future research. Third, our study did not address comorbidities of patients, such as ADHD or epilepsy, since the included studies did not provide relevant data. Fourth, we did not discriminate between Pearson’s correlation and Spearman’s correlation in the analyses, although they were technically different. Finally, the research included in this meta-analysis focused on the cross-sectional description of sleep problems in ASD, which only captures symptoms at a specific age and generally ignores developmental changes. In this regard, longitudinal studies examining the developmental changes in sleep problems, their links with behavioral problems and core symptoms of ASD, and what factors might be associated with this change may be needed. In addition, the identification of the sensory profile will help us to understand the causal relationship between special sensory experiences and sleep.

### Supplementary Information

Below is the link to the electronic supplementary material.

Supplementary file1 (DOCX 223 KB)

## Data Availability

The data used for this study cannot be presented online because included articles are protected by copyright. Additional data from our analysis can be shared by contacting either of the corresponding authors (Jae Il Shin; shinji@yuhs.ac, Keun-Ah Cheon; kacheon@yuhs.ac).
