# Supplementary material for: Correlations between sleep problems, core symptoms, and behavioral problems in children and adolescents with autism spectrum disorder: a systematic review and meta-analysis
Source: Eur Child Adolesc Psychiatry. 2023 Jul 21;33(5):1539–49. doi: 10.1007/s00787-023-02253-1 (PMC11098879; doi:10.1007/s00787-023-02253-1)
Supplement: Supplementary file 1 — Supplementary file1 (DOCX 223 KB) [file 787_2023_2253_MOESM1_ESM.docx]

**Supplementary materials**

**Correlations between sleep problems, core symptoms, and behavioral problems in children and adolescents with autism spectrum disorder: a systematic review and meta-analysis**

Heeyeon Kim^1,2†^, Jae Han Kim^3†^, Ju Hyeon Yi^3†^, Jong Yeob Kim^3^, Marco Solmi^4,5,6,7^, Samuele Cortese^8,9,10,11^, Lee Smith^12^, Ai Koyanagi^13,14^, Jae Il Shin^3,15*^, Keun-Ah Cheon^1,2*^, Paolo Fusar-Poli^16,17,18,19^

Index

[**eAppendix 1. Preferred Reporting Items for Systematic Reviews and Meta-analyses guidelines checklist** 4](#_Toc122008470)

[**eTable 1.1. Preferred Reporting Items for Systematic Reviews and Meta-analyses guidelines checklist** 4](#_Toc122008471)

[**eAppendix 2. Preferred Reporting Items for Systematic Reviews and Meta-analyses guidelines abstract checklist** 7](#_Toc122008472)

[**eTable 2.1. Preferred Reporting Items for Systematic Reviews and Meta-analyses guidelines abstract checklist** 7](#_Toc122008473)

[**eAppendix 3. Full search strategies (The last search was done on April 27^th^, 2022)** 8](#_Toc122008474)

[**eTable 3.1. Full search strategies** 8](#_Toc122008475)

[**eAppendix 4. The list of excluded articles by full text screening with exclusion reason** 9](#_Toc122008476)

[**eTable 4.1. The list of excluded articles by full text screening with exclusion reason (Search for correlation between sleep problems & ASD core symptoms)** 9](#_Toc122008477)

[**eTable 4.2. The list of excluded articles by full text screening with exclusion reason (Search for correlation between sleep problems & ASD behavioral problems)** 11](#_Toc122008478)

[**eAppendix 5. Clarification of sleep problems, ASD core symptoms, and ASD behavioral problems** 17](#_Toc122008479)

[**eTable 5.1. Unification of parameters for sleep problems** 18](#_Toc122008480)

[**eTable 5.2. Unification of parameters for ASD core symptoms** 19](#_Toc122008481)

[**eTable 5.3. Unification of parameters for behavioral problems** 20](#_Toc122008482)

[**eAppendix 6. The list of included studies** 21](#_Toc122008483)

[**eTable 6.1. The list of included studies (Search for correlations between specific sleep problems & ASD core symptoms)** 21](#_Toc122008484)

[**eTable 6.2. The list of included studies (Search for correlations between specific sleep problems & ASD behavioral problems)** 22](#_Toc122008485)

[**eTable 6.3. The list of included studies (Search for correlations between ASD core symptoms & ASD behavioral problems)** 23](#_Toc122008486)

[**eAppendix 7. Result of the study quality assessment (AXIS score)** 24](#_Toc122008487)

[**eTable 7.1. Result of the AXIS score** 24](#_Toc122008488)

[**eAppendix 8. Pooled correlations between sleep problems & ASD core symptoms** 26](#_Toc122008489)

[**eTable 8.1. Pooled correlations between sleep problems & ASD core symptoms** 26](#_Toc122008490)

[**eTable 8.2. Statistical results of meta-analyses between sleep problems & ASD core symptoms** 27](#_Toc122008491)

[**eTable 8.3. Effect size correction using the trim-and-fill method (Total sleep problem & Total core symptoms)** 29](#_Toc122008492)

[**eAppendix 9. Pooled correlations between sleep problems & behavioral problems** 30](#_Toc122008493)

[**eTable 9.1. Pooled correlations between sleep problems & behavioral problems** 30](#_Toc122008494)

[**eTable 9.2. Statistical results of meta-analyses between sleep problems & behavioral problems** 31](#_Toc122008495)

[**eTable 9.3. Effect size correction using the trim-and-fill method (Sleep anxiety & Aggressive/Delinquent problem)** 34](#_Toc122008496)

[**eTable 9.4. Effect size correction using the trim-and-fill method (Sleep duration & Aggressive/Delinquent problem)** 34](#_Toc122008497)

[**eAppendix 10. Pooled correlations between ASD core symptoms & behavioral problems** 35](#_Toc122008498)

[**eTable 10.1. Pooled correlations between ASD core symptoms & behavioral problems** 35](#_Toc122008499)

[**eTable 10.2. Statistical results of meta-analyses between ASD core symptoms & behavioral problems** 36](#_Toc122008500)

[**eTable 10.3. Effect size correction using the trim-and-fill method (Affective/Anxiety problem & Restricted and repetitive behavior)** 37](#_Toc122008501)

[**eAppendix 11. Statistical results of meta-regression (mean age of participants, percentage of boys, mean IQ, and AXIS score)** 38](#_Toc122008502)

[**eTable 11.1. Statistical results of meta-regression – sleep problems & ASD core symptoms** 38](#_Toc122008503)

[**eTable 11.2. Statistical results of meta-regression – sleep problems & behavioral problems** 41](#_Toc122008504)

[**eTable 11.3. Statistical results of meta-regression analyses - ASD core symptoms & behavioral problems** 44](#_Toc122008505)

[**eAppendix 12. Statistical results of the subgroup analyses - Medication use status (note that subgroup analysis for correlations between ASD core symptoms & behavioral problems was unavailable)** 46](#_Toc122008506)

[**eTable 12.1. Statistical results of the subgroup analyses – sleep problems & ASD core symptoms** 46](#_Toc122008507)

[**eTable 12.2. Statistical results of the subgroup analyses – sleep problems & behavioral problems** 48](#_Toc122008508)

# **eAppendix 1. Preferred Reporting Items for Systematic Reviews and Meta-analyses guidelines checklist**

## **eTable 1.1. Preferred Reporting Items for Systematic Reviews and Meta-analyses guidelines checklist**

| **Section and Topic** | **Item #** | **Checklist item** | **Location where item is reported** |
| --- | --- | --- | --- |
| **TITLE** | | |  |
| Title | 1 | Identify the report as a systematic review. | Title page |
| **ABSTRACT** | | |  |
| Abstract | 2 | See the PRISMA 2020 for Abstracts checklist. | Appendix p 8 |
| **INTRODUCTION** | | |  |
| Rationale | 3 | Describe the rationale for the review in the context of existing knowledge. | Manuscript p 4 |
| Objectives | 4 | Provide an explicit statement of the objective(s) or question(s) the review addresses. | Manuscript p 4 |
| **METHODS** | | |  |
| Eligibility criteria | 5 | Specify the inclusion and exclusion criteria for the review and how studies were grouped for the syntheses. | Manuscript p 5 |
| Information sources | 6 | Specify all databases, registers, websites, organisations, reference lists and other sources searched or consulted to identify studies. Specify the date when each source was last searched or consulted. | Manuscript p 5 |
| Search strategy | 7 | Present the full search strategies for all databases, registers and websites, including any filters and limits used. | Appendix p 9 |
| Selection process | 8 | Specify the methods used to decide whether a study met the inclusion criteria of the review, including how many reviewers screened each record and each report retrieved, whether they worked independently, and if applicable, details of automation tools used in the process. | Manuscript p 5 |
| Data collection process | 9 | Specify the methods used to collect data from reports, including how many reviewers collected data from each report, whether they worked independently, any processes for obtaining or confirming data from study investigators, and if applicable, details of automation tools used in the process. | Manuscript p 5 |
| Data items | 10a | List and define all outcomes for which data were sought. Specify whether all results that were compatible with each outcome domain in each study were sought (e.g. for all measures, time points, analyses), and if not, the methods used to decide which results to collect. | Manuscript p 5 |
|  | 10b | List and define all other variables for which data were sought (e.g. participant and intervention characteristics, funding sources). Describe any assumptions made about any missing or unclear information. | Manuscript p 5 |
| Study risk of bias assessment | 11 | Specify the methods used to assess risk of bias in the included studies, including details of the tool(s) used, how many reviewers assessed each study and whether they worked independently, and if applicable, details of automation tools used in the process. | Manuscript p 6 |
| Effect measures | 12 | Specify for each outcome the effect measure(s) (e.g. risk ratio, mean difference) used in the synthesis or presentation of results. | Manuscript p 6 |
| Synthesis methods | 13a | Describe the processes used to decide which studies were eligible for each synthesis (e.g. tabulating the study intervention characteristics and comparing against the planned groups for each synthesis (item #5)). | Manuscript p 5 |
|  | 13b | Describe any methods required to prepare the data for presentation or synthesis, such as handling of missing summary statistics, or data conversions. | Manuscript p 6 |
|  | 13c | Describe any methods used to tabulate or visually display results of individual studies and syntheses. | Manuscript pp 6-7 |
|  | 13d | Describe any methods used to synthesize results and provide a rationale for the choice(s). If meta-analysis was performed, describe the model(s), method(s) to identify the presence and extent of statistical heterogeneity, and software package(s) used. | Manuscript pp 6-7 |
|  | 13e | Describe any methods used to explore possible causes of heterogeneity among study results (e.g. subgroup analysis, meta-regression). | Manuscript p 7 |
|  | 13f | Describe any sensitivity analyses conducted to assess robustness of the synthesized results. | Manuscript p 7 |
| Reporting bias assessment | 14 | Describe any methods used to assess risk of bias due to missing results in a synthesis (arising from reporting biases). | Manuscript p 6 |
| Certainty assessment | 15 | Describe any methods used to assess certainty (or confidence) in the body of evidence for an outcome. | Non-applicable |
| **RESULTS** | | |  |
| Study selection | 16a | Describe the results of the search and selection process, from the number of records identified in the search to the number of studies included in the review, ideally using a flow diagram. | Figure 1, Manuscript p 8 |
|  | 16b | Cite studies that might appear to meet the inclusion criteria, but which were excluded, and explain why they were excluded. | Appendix pp 10-17 |
| Study characteristics | 17 | Cite each included study and present its characteristics. | Manuscript pp 8-9 |
| Risk of bias in studies | 18 | Present assessments of risk of bias for each included study. | Table 1, Manuscript p 10 |
| Results of individual studies | 19 | For all outcomes, present, for each study: (a) summary statistics for each group (where appropriate) and (b) an effect estimate and its precision (e.g. confidence/credible interval), ideally using structured tables or plots. | Table 1 |
| Results of syntheses | 20a | For each synthesis, briefly summarise the characteristics and risk of bias among contributing studies. | Figure 2, Manuscript pp 8-9 |
|  | 20b | Present results of all statistical syntheses conducted. If meta-analysis was done, present for each the summary estimate and its precision (e.g. confidence/credible interval) and measures of statistical heterogeneity. If comparing groups, describe the direction of the effect. | Figure 2, Manuscript pp 8-9, Appendix pp 26-38 |
|  | 20c | Present results of all investigations of possible causes of heterogeneity among study results. | Manuscript pp 9-10, Appendix pp 39-51 |
|  | 20d | Present results of all sensitivity analyses conducted to assess the robustness of the synthesized results. | Figure 3, Appendix pp 39-51 |
| Reporting biases | 21 | Present assessments of risk of bias due to missing results (arising from reporting biases) for each synthesis assessed. | Manuscript p 10 |
| Certainty of evidence | 22 | Present assessments of certainty (or confidence) in the body of evidence for each outcome assessed. | Non-applicable |
| **DISCUSSION** | | |  |
| Discussion | 23a | Provide a general interpretation of the results in the context of other evidence. | Manuscript p 11 |
|  | 23b | Discuss any limitations of the evidence included in the review. | Manuscript p 13 |
|  | 23c | Discuss any limitations of the review processes used. | Manuscript p 13 |
|  | 23d | Discuss implications of the results for practice, policy, and future research. | Manuscript p 13 |
| **OTHER INFORMATION** | | |  |
| Registration and protocol | 24a | Provide registration information for the review, including register name and registration number, or state that the review was not registered. | Manuscript p 5 |
|  | 24b | Indicate where the review protocol can be accessed, or state that a protocol was not prepared. | Manuscript p 5 |
|  | 24c | Describe and explain any amendments to information provided at registration or in the protocol. | Manuscript p 5 |
| Support | 25 | Describe sources of financial or non-financial support for the review, and the role of the funders or sponsors in the review. | Manuscript p 14 |
| Competing interests | 26 | Declare any competing interests of review authors. | Manuscript p 14 |
| Availability of data, code and other materials | 27 | Report which of the following are publicly available and where they can be found: template data collection forms; data extracted from included studies; data used for all analyses; analytic code; any other materials used in the review. | Manuscript p 14 |

# **eAppendix 2. Preferred Reporting Items for Systematic Reviews and Meta-analyses guidelines abstract checklist**

## **eTable 2.1. Preferred Reporting Items for Systematic Reviews and Meta-analyses guidelines abstract checklist**

| **Section and Topic** | **Item #** | **Checklist item** | **Reported (Yes/No)** |
| --- | --- | --- | --- |
| **TITLE** | | |  |
| Title | 1 | Identify the report as a systematic review. | Yes |
| **BACKGROUND** | | |  |
| Objectives | 2 | Provide an explicit statement of the main objective(s) or question(s) the review addresses. | Yes |
| **METHODS** | | |  |
| Eligibility criteria | 3 | Specify the inclusion and exclusion criteria for the review. | Yes |
| Information sources | 4 | Specify the information sources (e.g. databases, registers) used to identify studies and the date when each was last searched. | Yes |
| Risk of bias | 5 | Specify the methods used to assess risk of bias in the included studies. | Yes |
| Synthesis of results | 6 | Specify the methods used to present and synthesise results. | Yes |
| **RESULTS** | | |  |
| Included studies | 7 | Give the total number of included studies and participants and summarise relevant characteristics of studies. | Yes |
| Synthesis of results | 8 | Present results for main outcomes, preferably indicating the number of included studies and participants for each. If meta-analysis was done, report the summary estimate and confidence/credible interval. If comparing groups, indicate the direction of the effect (i.e. which group is favoured). | Yes |
| **DISCUSSION** | | |  |
| Limitations of evidence | 9 | Provide a brief summary of the limitations of the evidence included in the review (e.g. study risk of bias, inconsistency and imprecision). | Yes |
| Interpretation | 10 | Provide a general interpretation of the results and important implications. | Yes |
| **OTHER** | | |  |
| Funding | 11 | Specify the primary source of funding for the review. | No |
| Registration | 12 | Provide the register name and registration number. | Yes |

# **eAppendix 3. Full search strategies (The last search was done on April 27^th^, 2022)**

## **eTable 3.1. Full search strategies**

| **Keywords** | | | |
| --- | --- | --- | --- |
| **AUTISM SPECTRUM DISORDERS** | (autis* OR Asperg* OR (pervasive developmental disorder)) | | |
| **SLEEP PROBLEMS** | (polysomnograph* OR actigraph* OR CSHQ OR (children's sleep habits questionnaire) OR sleep* OR (sleep problem) OR (sleep quality) OR (sleep disturbance) OR (sleep disruption) OR (sleep apnea) OR (sleep deprivation) OR (sleep duration) OR (short sleep) OR (long sleep) OR (chronotype) OR (eveningness) OR (circadian misalignment) OR sleepiness OR alertness OR vigilance OR insomnia) | | |
| **BEHAVIORAL PROBLEMS** | (Irritability OR agitation OR aggression OR (temper tantrums) OR (self-injurious behavior) OR (problem behavior) OR (attention deficit and disruptive behavior disorders) OR (self-injurious behavior) OR CBCL OR (child behavior checklist) OR ABC OR (Autism Behavior Checklist) OR (Aberrant Behavior Checklist)) | | |
| **CORE SYMTOMS OF AUTISM SPECTRUM DISORDERS** | (restrict* OR repetit* OR social OR ADOS OR (Autism Diagnostic Observation Schedule) OR ADI OR (autism diagnostic interview) OR ADI OR (autism diagnostic interview AND revised) OR CARS OR (childhood autism rating scale) OR SRS OR (social responsiveness scale)) | | |
| **NOT** | (therapy OR intervention OR treatment OR review OR meta-analysis OR (fragile X syndrome) OR (Rett syndrome) OR (mouse model)) | | |
| **Search results** | | | |
| **SLEEP** AND **AUTISM CORE** AND **NOT** | | **SLEEP** AND **BEHAVIOR** AND **NOT** | **AUTISM CORE** AND **BEHAVIOR** AND **NOT** |
| PubMed (166 articles were found) | | PubMed (104 articles were found) | PubMed (1006 articles were found) |
| Scopus (458 articles were found) | | Scopus (366 articles were found) | Scopus (2232 articles were found) |
| Web of science (336 articles were found) | | Web of science (275 articles were found) | Web of science (1785 articles were found) |
| Two articles regarding correlation between autism spectrum disorder core symptoms and behavioral problems were identified via references of relevants studies. | | | |

# **eAppendix 4. The list of excluded articles by full text screening with exclusion reason**

## **eTable 4.1. The list of excluded articles by full text screening with exclusion reason (Search for correlation between sleep problems & ASD core symptoms)**

| Adams 2014 [1] | did not investigate correlations of interest |
| --- | --- |
| Dewrang 2010 [2] | did not investigate correlations of interest |
| Dovgan 2019 [3] | did not investigate correlations of interest |
| Fadini 2015 [4] | did not investigate correlations of interest |
| Johansson 2018 [5] | did not investigate correlations of interest |
| Köse 2017 [6] | did not investigate correlations of interest |
| Krakowiak 2008 [7] | did not investigate correlations of interest |
| Lambert 2016 [8] | did not investigate correlations of interest |
| Matson 2011 [9] | did not investigate correlations of interest |
| Mazurek 2016 [10] | did not investigate correlations of interest |
| Patzold 1998 [11] | did not investigate correlations of interest |
| Türkoğlu 2021 [12] | did not investigate correlations of interest |
| Veatch 2017 [13] | did not investigate correlations of interest |
| Anders 2012 [14] | did not provide the outcome as correlation coefficients |
| DeVincent 2007 [15] | did not provide the outcome as correlation coefficients |
| MacDuffie 2020 [16] | did not provide the outcome as correlation coefficients |
| Miano 2007 [17] | did not provide the outcome as correlation coefficients |
| Romeo 2021 [18] | did not provide the outcome as correlation coefficients |
| Sadikova 2022 [19] | did not provide the outcome as correlation coefficients |
| Sikora 2012 [20] | did not provide the outcome as correlation coefficients |
| Verhoeff 2018 [21] | did not provide the outcome as correlation coefficients |
| Yavuz-Kodat 2020 [22] | did not provide the outcome as correlation coefficients |
| Elkhatib Smidt 2022 [23] | included participants over the age of 18 |
| Phung 2017 [24] | included participants over the age of 18 |
| May 2015 [25] | included participants without ASD |
| Park 2012 [26] | included participants without ASD |
| Saenz 2015 [27] | included participants without ASD |

1. Adams, H.L., J.L. Matson, and J. Jang, The relationship between sleep problems and challenging behavior among children and adolescents with autism spectrum disorder. Research in Autism Spectrum Disorders, 2014. **8**(9): p. 1024-1030.

2. Dewrang, P. and A.D. Sandberg, Parental retrospective assessment of development and behavior in Asperger syndrome during the first 2 years of life. Research in Autism Spectrum Disorders, 2010. **4**(3): p. 461-473.

3. Dovgan, K., M.O. Mazurek, and J. Hansen, Measurement invariance of the child behavior checklist in children with autism spectrum disorder with and without intellectual disability: Follow-up study. Research in Autism Spectrum Disorders, 2019. **58**: p. 19-29.

4. Fadini, C.C., et al., Influence of sleep disorders on the behavior of individuals with autism spectrum disorder. Frontiers in Human Neuroscience, 2015. **9**: p. 347.

5. Johansson, A.E., et al., Characteristics of sleep in children with autism spectrum disorders from the Simons Simplex Collection. Research in Autism Spectrum Disorders, 2018. **53**: p. 18-30.

6. Köse, S., et al., Sleep problems in children with autism spectrum disorder and intellectual disability without autism spectrum disorder. Sleep Medicine, 2017. **40**: p. 69-77.

7. Krakowiak, P., et al., Sleep problems in children with autism spectrum disorders, developmental delays, and typical development: A population‐based study. Journal of sleep research, 2008. **17**(2): p. 197-206.

8. Lambert, A., et al., Poor sleep affects daytime functioning in typically developing and autistic children not complaining of sleep problems: A questionnaire-based and polysomnographic study. Research in autism spectrum disorders, 2016. **23**: p. 94-106.

9. Matson, J.L., et al., Effects of symptoms of co-morbid psychopathology on challenging behaviours among infants and toddlers with Autistic Disorder and PDD-NOS as assessed with the Baby and Infant Screen for Children with aUtIsm Traits (BISCUIT). Developmental neurorehabilitation, 2011. **14**(3): p. 129-139.

10. Mazurek, M.O. and K. Sohl, Sleep and behavioral problems in children with autism spectrum disorder. Journal of autism and developmental disorders, 2016. **46**(6): p. 1906-1915.

11. Patzold, L., A. Richdale, and B. Tonge, An investigation into sleep characteristics of children with autism and Asperger’s disorder. Journal of paediatrics and child health, 1998. **34**(6): p. 528-533.

12. Türkoğlu, S., et al., The relationship between irritability and autism symptoms in children with ASD in COVID‐19 home confinement period. International Journal of Clinical Practice, 2021. **75**(11): p. e14742.

13. Veatch, O.J., et al., Shorter sleep duration is associated with social impairment and comorbidities in ASD. Autism Research, 2017. **10**(7): p. 1221-1238.

14. Anders, T., et al., Sleep and daytime functioning: a short-term longitudinal study of three preschool-age comparison groups. American Journal on Intellectual and Developmental Disabilities, 2012. **117**(4): p. 275-290.

15. DeVincent, C.J., et al., Sleep disturbance and its relation to DSM-IV psychiatric symptoms in preschool-age children with pervasive developmental disorder and community controls. Journal of Child Neurology, 2007. **22**(2): p. 161-169.

16. MacDuffie, K.E., et al., Sleep problems and trajectories of restricted and repetitive behaviors in children with neurodevelopmental disabilities. Journal of Autism and Developmental Disorders, 2020. **50**(11): p. 3844-3856.

17. Miano, S., et al., Sleep in children with autistic spectrum disorder: a questionnaire and polysomnographic study. Sleep medicine, 2007. **9**(1): p. 64-70.

18. Romeo, D.M., et al., Sleep disorders in autism spectrum disorder pre-school children: an evaluation using the sleep disturbance scale for children. Medicina, 2021. **57**(2): p. 95.

19. Sadikova, E., K. Dovgan, and M.O. Mazurek, Longitudinal Examination of Sleep Problems and Symptom Severity in Children with Autism Spectrum Disorder. Journal of Autism and Developmental Disorders, 2022: p. 1-9.

20. Sikora, D.M., et al., The relationship between sleep problems and daytime behavior in children of different ages with autism spectrum disorders. Pediatrics, 2012. **130**(Supplement_2): p. S83-S90.

21. Verhoeff, M.E., et al., The bidirectional association between sleep problems and autism spectrum disorder: a population-based cohort study. Molecular Autism, 2018. **9**(1): p. 1-9.

22. Yavuz-Kodat, E., et al., Disturbances of continuous sleep and circadian rhythms account for behavioral difficulties in children with autism spectrum disorder. Journal of clinical medicine, 2020. **9**(6): p. 1978.

23. Elkhatib Smidt, S.D., et al., The relationship between autism spectrum and sleep–wake traits. Autism Research, 2022. **15**(4): p. 641-652.

24. Phung, J.N. and W.A. Goldberg, Poor sleep quality is associated with discordant peer relationships among adolescents with Autism Spectrum Disorder. Research in Autism Spectrum Disorders, 2017. **34**: p. 10-18.

25. May, T., et al., Sleep in high-functioning children with autism: longitudinal developmental change and associations with behavior problems. Behavioral sleep medicine, 2015. **13**(1): p. 2-18.

26. Park, S., et al., Sleep problems and their correlates and comorbid psychopathology of children with autism spectrum disorders. Research in Autism Spectrum Disorders, 2012. **6**(3): p. 1068-1072.

27. Saenz, J., A. Yaugher, and G.M. Alexander, Sleep in infancy predicts gender specific social-emotional problems in toddlers. Frontiers in Pediatrics, 2015. **3**: p. 42.

## **eTable 4.2. The list of excluded articles by full text screening with exclusion reason (Search for correlation between sleep problems & ASD behavioral problems)**

| Al Backer 2018 [1] | did not investigate correlations of interest |
| --- | --- |
| Anders 2012 [2] | did not investigate correlations of interest |
| DeVincent 2007 [3] | did not investigate correlations of interest |
| Gunes 2019 [4] | did not investigate correlations of interest |
| Lambert 2016 [5] | did not investigate correlations of interest |
| Phung 2017 [6] | did not investigate correlations of interest |
| Richdale 2015 [7] | did not investigate correlations of interest |
| Saré 2020 [8] | did not investigate correlations of interest |
| Taylor 2021 [9] | did not investigate correlations of interest |
| Türkoğlu 2021 [10] | did not investigate correlations of interest |
| Adams 2014 [11] | did not provide the outcome as correlation coefficients |
| Callahan 2021 [12] | did not provide the outcome as correlation coefficients |
| Goodlin-Jones 2009 [13] | did not provide the outcome as correlation coefficients |
| Johnson 2018 [14] | did not provide the outcome as correlation coefficients |
| Kelmanson 2020 [15] | did not provide the outcome as correlation coefficients |
| Li 2019 [16] | did not provide the outcome as correlation coefficients |
| Samanta 2020 [17] | did not provide the outcome as correlation coefficients |
| Sikora 2012 [18] | did not provide the outcome as correlation coefficients |
| Veatch 2017 [19] | did not provide the outcome as correlation coefficients |
| Yavuz-Kodat 2020 [20] | did not provide the outcome as correlation coefficients |
| May 2015 [21] | included participants without ASD |
| Adams 2014 [22] | included participants without ASD |
| Castelnovo 2021 [23] | included participants without ASD |
| Horiuchi 2020 [24] | included participants without ASD |
| Kara 2019 [25] | included participants without ASD |
| Lee 2021 [26] | included participants without ASD |
| Levin 2016 [27] | included participants without ASD |
| MacDuffie 2020 [28] | included participants without ASD |
| Park 2012 [29] | included participants without ASD |
| Scheithauer 2015 [30] | included participants without ASD |
| Uren 2019 [31] | included participants without ASD |
| Verhoeff 2018 [32] | included participants without ASD |
| Yang 2021 [33] | included participants without ASD |
| Haney 2014 [34] | not an original article |
| Richdale 2018 [35] | not an original article |

1. Al Backer, N.B., et al., *The relationship between sleep and cognitive performance in Autism Spectrum Disorder (ASD): a pilot study.* Children, 2018. **5**(11): p. 153.

2. Anders, T., et al., *Sleep and daytime functioning: a short-term longitudinal study of three preschool-age comparison groups.* American Journal on Intellectual and Developmental Disabilities, 2012. **117**(4): p. 275-290.

3. DeVincent, C.J., et al., *Sleep disturbance and its relation to DSM-IV psychiatric symptoms in preschool-age children with pervasive developmental disorder and community controls.* Journal of Child Neurology, 2007. **22**(2): p. 161-169.

4. Gunes, S., et al., *Sleep problems in children with autism spectrum disorder: clinical correlates and the impact of attention deficit hyperactivity disorder.* Neuropsychiatric disease and treatment, 2019. **15**: p. 763.

5. Lambert, A., et al., *Poor sleep affects daytime functioning in typically developing and autistic children not complaining of sleep problems: A questionnaire-based and polysomnographic study.* Research in autism spectrum disorders, 2016. **23**: p. 94-106.

6. Phung, J.N. and W.A. Goldberg, *Poor sleep quality is associated with discordant peer relationships among adolescents with Autism Spectrum Disorder.* Research in Autism Spectrum Disorders, 2017. **34**: p. 10-18.

7. Richdale, A.L. and C.L. Baglin, *Self-report and caregiver-report of sleep and psychopathology in children with high-functioning autism spectrum disorder: a pilot study.* Developmental Neurorehabilitation, 2015. **18**(4): p. 272-279.

8. Saré, R.M. and C.B. Smith, *Association between sleep deficiencies with behavioral problems in autism spectrum disorder: subtle sex differences.* Autism Research, 2020. **13**(10): p. 1802-1810.

9. Taylor, B.J., C.F. Reynolds III, and M. Siegel, *Insomnia subtypes and clinical impairment in hospitalized children with autism spectrum disorder.* Autism, 2021. **25**(3): p. 656-666.

10. Türkoğlu, S., et al., *The relationship between irritability and autism symptoms in children with ASD in COVID‐19 home confinement period.* International Journal of Clinical Practice, 2021. **75**(11): p. e14742.

11. Adams, H.L., J.L. Matson, and J. Jang, *The relationship between sleep problems and challenging behavior among children and adolescents with autism spectrum disorder.* Research in Autism Spectrum Disorders, 2014. **8**(9): p. 1024-1030.

12. Callahan, M., et al., *Aggression in Toddlers with Autism Spectrum Disorder as Predicted by Sleep Problems.* Journal of Developmental and Physical Disabilities, 2022. **34**(4): p. 645-654.

13. Goodlin‐Jones, B., et al., *Sleep problems, sleepiness and daytime behavior in preschool‐age children.* Journal of Child Psychology and Psychiatry, 2009. **50**(12): p. 1532-1540.

14. Johnson, C.R., et al., *Exploring sleep quality of young children with autism spectrum disorder and disruptive behaviors.* Sleep medicine, 2018. **44**: p. 61-66.

15. Kelmanson, I.A., *Sleep disturbances and their associations with emotional/behavioural problems in 5-year-old boys with autism spectrum disorders.* Early Child Development and Care, 2018.

16. LI, Y.-Y., *Association between behavioral problems and sleep problems among children with autism spectrum disorder.* Journal of Shanghai Jiaotong University (Medical Science), 2019: p. 505-509.

17. Samanta, P., et al., *Sleep disturbances and associated factors among 2-6-year-old male children with autism in Bhubaneswar, India.* Sleep Medicine, 2020. **67**: p. 77-82.

18. Sikora, D.M., et al., *The relationship between sleep problems and daytime behavior in children of different ages with autism spectrum disorders.* Pediatrics, 2012. **130**(Supplement_2): p. S83-S90.

19. Veatch, O.J., et al., *Shorter sleep duration is associated with social impairment and comorbidities in ASD.* Autism Research, 2017. **10**(7): p. 1221-1238.

20. Yavuz-Kodat, E., et al., *Disturbances of continuous sleep and circadian rhythms account for behavioral difficulties in children with autism spectrum disorder.* Journal of clinical medicine, 2020. **9**(6): p. 1978.

21. May, T., et al., *Sleep in high-functioning children with autism: longitudinal developmental change and associations with behavior problems.* Behavioral sleep medicine, 2015. **13**(1): p. 2-18.

22. Adams, H.L., et al., *The relationship between autism symptom severity and sleep problems: Should bidirectionality be considered?* Research in Autism Spectrum Disorders, 2014. **8**(3): p. 193-199.

23. Castelnovo, A., et al., *Behavioural and emotional profiles of children and adolescents with disorders of arousal.* Journal of sleep research, 2021. **30**(1): p. e13188.

24. Horiuchi, F., et al., *The association between autistic traits and sleep habits/problems in toddlers.* Developmental Neuropsychology, 2020. **45**(7-8): p. 485-495.

25. Kara, T., et al., *Sleep habits as an indicator of social competence and behaviour in pre-schoolers in the context of neurodevelopmental disorders.* Psychiatry and Clinical Psychopharmacology, 2019. **29**(1): p. 68-75.

26. Lee, T., et al., *Sleep difficulties and related behavioral problems in Korean preschool children.* Sleep Medicine, 2021. **87**: p. 119-126.

27. Levin, A. and A. Scher, *Sleep problems in young children with autism spectrum disorders: A study of parenting stress, mothers' sleep‐related cognitions, and bedtime behaviors.* CNS neuroscience & therapeutics, 2016. **22**(11): p. 921-927.

28. MacDuffie, K.E., et al., *Sleep problems and trajectories of restricted and repetitive behaviors in children with neurodevelopmental disabilities.* Journal of Autism and Developmental Disorders, 2020. **50**(11): p. 3844-3856.

29. Park, S., et al., *Sleep problems and their correlates and comorbid psychopathology of children with autism spectrum disorders.* Research in Autism Spectrum Disorders, 2012. **6**(3): p. 1068-1072.

30. Scheithauer, M.C. and J. Zarcone, *Evaluating the relationship between sleep and problem behavior in children with disabilities.* Behavior Analysis in Practice, 2015. **8**(1): p. 27-36.

31. Uren, J., et al., *Sleep problems and anxiety from 2 to 8 years and the influence of autistic traits: a longitudinal study.* European Child & Adolescent Psychiatry, 2019. **28**(8): p. 1117-1127.

32. Verhoeff, M.E., et al., *The bidirectional association between sleep problems and autism spectrum disorder: a population-based cohort study.* Molecular Autism, 2018. **9**(1): p. 1-9.

33. Yang, L., et al., *Analysis of behavioral problems in children with sleep-disordered breathing and decreased REM sleep.* International Journal of Pediatric Otorhinolaryngology, 2021. **147**: p. 110783.

34. Haney, T. and K.M. Kott, *Sleep problems in children: An overlooked factor in evaluating behavior.* Journal of Psychosocial Nursing and Mental Health Services, 2014. **52**(10): p. 27-32.

35. Richdale, A. and S. Roussis. *Sleep problem severity and behaviour in children with autism aged 2-to 5-years*. in *JOURNAL OF SLEEP RESEARCH*. 2018. WILEY 111 RIVER ST, HOBOKEN 07030-5774, NJ USA.

36. Schreck, K., et al. *Possible relationships among daytime behaviour, mental health and sleep problems for children with autism spectrum disorder*. in *JOURNAL OF INTELLECTUAL DISABILITY RESEARCH*. 2016. WILEY-BLACKWELL 111 RIVER ST, HOBOKEN 07030-5774, NJ USA.

37. Tzischinsky, O., et al. *Children with autism who have sleep problems exhibit abnormally high sensory sensitivities*. in *JOURNAL OF SLEEP RESEARCH*. 2016. WILEY-BLACKWELL 111 RIVER ST, HOBOKEN 07030-5774, NJ USA.

**eTable 4.3. The list of excluded articles by full text screening with exclusion reason (Search for correlation between ASD behavioral problems & ASD core symptoms)**

| Anagnostou 2011 [1] | did not investigate correlations of interest |
| --- | --- |
| Baeza-Velasco 2014 [2] | did not investigate correlations of interest |
| Baribeau 2021 [3] | did not investigate correlations of interest |
| Davidsson 2017 [4] | did not investigate correlations of interest |
| De Giacomo 2016 [5] | did not investigate correlations of interest |
| Dovgan 2019 [6] | did not investigate correlations of interest |
| Downs 2004 [7] | did not investigate correlations of interest |
| Duerden 2014 [8] | did not investigate correlations of interest |
| Faja 2019 [9] | did not investigate correlations of interest |
| Gadow 2016 [10] | did not investigate correlations of interest |
| Georgiades 2011 [11] | did not investigate correlations of interest |
| Goldsmith 2018 [12] | did not investigate correlations of interest |
| Hesapçioǧlu 2012 [13] | did not investigate correlations of interest |
| Jamal 2021 [14] | did not investigate correlations of interest |
| Kerns 2021 [15] | did not investigate correlations of interest |
| Miranda 2020 [16] | did not investigate correlations of interest |
| Pugliese 2013 [17] | did not investigate correlations of interest |
| Rescorla 2019 [18] | did not investigate correlations of interest |
| Richler 2007 [19] | did not investigate correlations of interest |
| Turygin 2013 [20] | did not investigate correlations of interest |
| Waters 2012 [21] | did not investigate correlations of interest |
| Westerveld 2021 [22] | did not investigate correlations of interest |
| Chaxiong 2022 [23] | did not provide the outcome as correlation coefficients |
| Gotham 2013 [24] | did not provide the outcome as correlation coefficients |
| Lance 2014 [25] | did not provide the outcome as correlation coefficients |
| Menezes 2021 [26] | did not provide the outcome as correlation coefficients |
| Park 2012 [27] | did not provide the outcome as correlation coefficients |
| Samson 2014 [28] | did not provide the outcome as correlation coefficients |
| Werkman 2020 [29] | did not provide the outcome as correlation coefficients |
| Brinkley 2007 [30] | included participants over the age of 18 |
| Fink 2018 [31] | included participants over the age of 18 |
| Richards 2017 [32] | included participants over the age of 18 |
| Chadwick 2008 [33] | included participants without ASD |
| Dworzynski 2009 [34] | included participants without ASD |
| Frazier 2014 [35] | included participants without ASD |
| Jones 2009 [36] | included participants without ASD |
| Kaartinen 2019 [37] | included participants without ASD |
| Noordhof 2015 [38] | included participants without ASD |
| Reid 2020 [39] | included participants without ASD |
| Sanz-Cervera 2015 [40] | included participants without ASD |
| Sprenger 2013 [41] | included participants without ASD |
| Duerden 2012 [42] | not an original article |
| Veenstra-VanderWeele 2011 [43] | not an original article |

1. Anagnostou, E., et al., *Factor analysis of repetitive behaviors in autism as measured by the Y-BOCS.* The Journal of neuropsychiatry and clinical neurosciences, 2011. **23**(3): p. 332-339.

2. Baeza-Velasco, C., et al., *Are aberrant behavioral patterns associated with the adaptive behavior trajectories of teenagers with autism spectrum disorders?* Research in Autism Spectrum Disorders, 2014. **8**(3): p. 304-311.

3. Baribeau, D.A., et al., *Co-occurring trajectories of anxiety and insistence on sameness behaviour in autism spectrum disorder.* The British Journal of Psychiatry, 2021. **218**(1): p. 20-27.

4. Davidsson, M., et al., *Anxiety and depression in adolescents with ADHD and autism spectrum disorders; correlation between parent-and self-reports and with attention and adaptive functioning.* Nordic Journal of Psychiatry, 2017. **71**(8): p. 614-620.

5. De Giacomo, A., et al., *Aggressive behaviors and verbal communication skills in autism spectrum disorders.* Global pediatric health, 2016. **3**: p. 2333794X16644360.

6. Dovgan, K.N. and M.O. Mazurek, *Relations among activity participation, friendship, and internalizing problems in children with autism spectrum disorder.* Autism, 2019. **23**(3): p. 750-758.

7. Downs, A. and T. Smith, *Emotional understanding, cooperation, and social behavior in high-functioning children with autism.* Journal of autism and developmental disorders, 2004. **34**(6): p. 625-635.

8. Duerden, E.G., et al., *Self-injurious behaviours are associated with alterations in the somatosensory system in children with autism spectrum disorder.* Brain structure and function, 2014. **219**(4): p. 1251-1261.

9. Faja, S. and L. Nelson Darling, *Variation in restricted and repetitive behaviors and interests relates to inhibitory control and shifting in children with autism spectrum disorder.* Autism, 2019. **23**(5): p. 1262-1272.

10. Gadow, K.D., et al., *Clinical correlates of co-occurring psychiatric and autism spectrum disorder (ASD) symptom-induced impairment in children with ASD.* Journal of abnormal child psychology, 2016. **44**(1): p. 129-139.

11. Georgiades, S., et al., *Phenotypic overlap between core diagnostic features and emotional/behavioral problems in preschool children with autism spectrum disorder.* Journal of autism and developmental disorders, 2011. **41**(10): p. 1321-1329.

12. Goldsmith, S.F. and E. Kelley, *Associations between emotion regulation and social impairment in children and adolescents with autism spectrum disorder.* Journal of autism and developmental disorders, 2018. **48**(6): p. 2164-2173.

13. Tural Hesapçıoğlu, S., et al., *Evaluation of the cases with autism spectrum disorders in terms of autism severity, their psychosocial and cognitive characteristic.* Yeni Symposium, 2012. **50**: p. 237-241.

14. Jamal, W., et al., *Reduced sensory habituation in autism and its correlation with behavioral measures.* Journal of autism and developmental disorders, 2021. **51**(9): p. 3153-3164.

15. Kerns, C.M., et al., *Clinically significant anxiety in children with autism spectrum disorder and varied intellectual functioning.* Journal of Clinical Child & Adolescent Psychology, 2021. **50**(6): p. 780-795.

16. Miranda, A., et al., *Relationships between the social communication questionnaire and pragmatic language, socialization skills, and behavioral problems in children with autism spectrum disorders.* Applied Neuropsychology: Child, 2020. **9**(2): p. 141-152.

17. Pugliese, C.E., et al., *Social anxiety predicts aggression in children with ASD: Clinical comparisons with socially anxious and oppositional youth.* Journal of Autism and Developmental Disorders, 2013. **43**(5): p. 1205-1213.

18. Rescorla, L.A., et al., *Structure, longitudinal invariance, and stability of the child behavior checklist 1½–5’s diagnostic and statistical manual of mental disorders–autism spectrum disorder scale: Findings from generation R (Rotterdam).* Autism, 2019. **23**(1): p. 223-235.

19. Richler, J., et al., *Restricted and repetitive behaviors in young children with autism spectrum disorders.* Journal of autism and developmental disorders, 2007. **37**(1): p. 73-85.

20. Turygin, N.C., et al., *The effect of DSM-5 criteria on externalizing, internalizing, behavioral and adaptive symptoms in children diagnosed with autism.* Developmental neurorehabilitation, 2013. **16**(4): p. 277-282.

21. Waters, P. and O. Healy, *Investigating the relationship between self-injurious behavior, social deficits, and cooccurring behaviors in children and adolescents with autism spectrum disorder.* Autism Research and Treatment, 2012. **2012**.

22. Westerveld, M.F., J. Paynter, and D. Adams, *Brief Report: Associations Between Autism Characteristics, Written and Spoken Communication Skills, and Social Interaction Skills in Preschool-Age Children on the Autism Spectrum.* Journal of Autism and Developmental Disorders, 2021. **51**(12): p. 4692-4697.

23. Chaxiong, P., et al., *Relations of restricted and repetitive behaviors to social skills in toddlers with autism.* Journal of autism and developmental disorders, 2022. **52**(4): p. 1423-1434.

24. Gotham, K., et al., *Exploring the relationship between anxiety and insistence on sameness in autism spectrum disorders.* Autism Research, 2013. **6**(1): p. 33-41.

25. Lance, E.I., et al., *Association between regression and self injury among children with autism.* Research in Developmental Disabilities, 2014. **35**(2): p. 408-413.

26. Menezes, M. and M.O. Mazurek, *Associations between domains of health-related quality of life and co-occurring emotional and behavioral problems in youth with autism spectrum disorder.* Research in Autism Spectrum Disorders, 2021. **82**: p. 101740.

27. Park, C.J., et al., *Brief report: The relationship between language skills, adaptive behavior, and emotional and behavior problems in pre-schoolers with autism.* Journal of Autism and Developmental Disorders, 2012. **42**(12): p. 2761-2766.

28. Samson, A.C., et al., *Emotion dysregulation and the core features of autism spectrum disorder.* Journal of Autism and developmental Disorders, 2014. **44**(7): p. 1766-1772.

29. Werkman, M., et al., *The moderating effect of cognitive abilities on the association between sensory processing and emotional and behavioural problems and social participation in autistic individuals.* Research in autism spectrum disorders, 2020. **78**: p. 101663.

30. Brinkley, J., et al., *Factor analysis of the aberrant behavior checklist in individuals with autism spectrum disorders.* Journal of autism and developmental disorders, 2007. **37**(10): p. 1949-1959.

31. Fink, E., et al., *Bullying-related behaviour in adolescents with autism: Links with autism severity and emotional and behavioural problems.* Autism, 2018. **22**(6): p. 684-692.

32. Richards, C., L. Davies, and C. Oliver, *Predictors of self-injurious behavior and self-restraint in autism spectrum disorder: Towards a hypothesis of impaired behavioral control.* Journal of Autism and Developmental Disorders, 2017. **47**(3): p. 701-713.

33. Chadwick, O., Y. Kusel, and M. Cuddy, *Factors associated with the risk of behaviour problems in adolescents with severe intellectual disabilities.* Journal of Intellectual Disability Research, 2008. **52**(10): p. 864-876.

34. Dworzynski, K., et al., *Relationship between symptom domains in autism spectrum disorders: a population based twin study.* Journal of Autism and Developmental Disorders, 2009. **39**(8): p. 1197-1210.

35. Frazier, T.W., et al., *Demographic and clinical correlates of autism symptom domains and autism spectrum diagnosis.* Autism, 2014. **18**(5): p. 571-582.

36. Jones, A.P., et al., *Phenotypic and aetiological associations between psychopathic tendencies, autistic traits, and emotion attribution.* Criminal Justice and Behavior, 2009. **36**(11): p. 1198-1212.

37. Kaartinen, M., et al., *Associations between cooperation, reactive aggression and social impairments among boys with autism spectrum disorder.* Autism, 2019. **23**(1): p. 154-166.

38. Noordhof, A., et al., *Integrating autism-related symptoms into the dimensional internalizing and externalizing model of psychopathology. The TRAILS Study.* Journal of abnormal child psychology, 2015. **43**(3): p. 577-587.

39. Reid, K.B., et al., *The association between social emotional development and symptom presentation in autism spectrum disorder.* Development and Psychopathology, 2020. **32**(4): p. 1206-1216.

40. Sanz-Cervera, P., et al., *Sensory processing in children with Autism Spectrum Disorder: Relationship with non-verbal IQ, autism severity and Attention Deficit/Hyperactivity Disorder symptomatology.* Research in Developmental Disabilities, 2015. **45**: p. 188-201.

41. Sprenger, L., et al., *Impact of ADHD symptoms on autism spectrum disorder symptom severity.* Research in developmental disabilities, 2013. **34**(10): p. 3545-3552.

42. Duerden, E.G., P. Szatmari, and S.W. Roberts, *Toward a better understanding of self injurious behaviors in children and adolescents with autism spectrum disorders.* Journal of autism and developmental disorders, 2012. **42**(11): p. 2515-2518.

43. Veenstra-VanderWeele, J. and Z. Warren, *Social communication deficits in the general population: how far out does the autism spectrum go?* Journal of the American Academy of Child and Adolescent Psychiatry, 2011. **50**(4): p. 326.

# **eAppendix 5. Clarification of sleep problems, ASD core symptoms, and ASD behavioral problems**

To assess sleep problems in ASD, we adopted the Children’s Sleep Habits Questionnaire (CSHQ) as a basic form of sleep problem because it has been commonly used to examine sleep problems in children and adolescents with ASD. It yields a total score of sleep problems and specific subscales of sleep problems, including bedtime resistance, daytime sleepiness, night waking, parasomnias, sleep anxiety, sleep duration, sleep-disordered breathing, and sleep-onset delay. To collect all published evidence, specific subscales of sleep problems in other questionnaires, such as the Sleep Disturbance Scale for Children (SDSC), were unified based on similarity (details in Appendix p. 19).

In terms of ASD core symptoms, based on the diagnostic criteria for ASD in DSM-5, two major symptoms (social communication problems and restricted and repetitive behavior) and total core symptoms were used as parameters of ASD core symptoms. For example, social affect severity, restricted and repetitive behavior severity, and calibrated severity score of the ADOS were unified into a social communication problem, restricted and repetitive behavior, and total core symptoms, respectively. Detailed information on the unification of parameters for ASD core symptoms is displayed in Appendix p. 20.

Finally, behavioral problems were assessed based on the total score of the Child Behavior Checklist (CBCL) and its syndrome subscales (affective/anxiety problems, somatic complaints, anxious/depressive problems, thought problems, attention problems, aggressive/delinquent problems, internalizing problems, and externalizing problems). Detailed information on the unification of parameters for ASD behavioral problems is displayed in Appendix p. 21.

We note that the abovementioned measures for sleep problems, ASD core symptoms, and ASD behavioral problems were determined through parameter unification after data extraction; hence, their designations might be different from the original ones. Parameter unification was mainly performed by a child and adolescent psychiatrist (HY; the first author) with the assistance of the co-first authors (JHK and JHY) via inspection of each question of each identified questionnaire for sleep problems, ASD core symptoms, and ASD behavioral problems to assess similarities. Through this, we anticipated that published evidence would be collated maximally while reducing heterogeneity.

## **eTable 5.1. Unification of parameters for sleep problems**

| Sleep measurements | Original parameters | Used as original | United to |
| --- | --- | --- | --- |
| CSHQ | Bedtime resistance | Bedtime resistance |  |
|  | Daytime sleepiness/Morning wakening |  | Daytime sleepiness |
|  | Night waking | Night waking |  |
|  | Parasomnias | Parasomnias |  |
|  | Sleep anxiety | Sleep anxiety |  |
|  | Sleep disordered breathing | Sleep disordered breathing |  |
|  | Sleep duration | Sleep duration |  |
|  | Sleep latency | Sleep latency |  |
|  | Total score |  | Total sleep problem |
| SDSC | Disorders in maintaining and initiating sleep |  | Daytime sleepiness |
|  | Disorders of arousal |  | Parasomnias |
|  | Disorders of excessive somnolence |  | Daytime sleepiness |
|  | Sleep breathing disorder |  | Sleep disordered breathing |
|  | Total score |  | Total sleep problem |
| PSQ | Breathing problems |  | Sleep disordered breathing |
|  | Insomnia |  | Sleep onset delay |
|  | Sleepiness |  | Daytime sleepiness |
|  | Total score |  | Total sleep problem |
| JSQ-P | Daytime excessive sleepiness |  | Daytime sleepiness |
|  | Obstructive sleep apnea |  | Sleep disordered breathing |
|  | Parasomnias | Parasomnias |  |
|  | Total score |  | Total sleep problem |
| SLEEP HABIT Survey | Daytime sleepiness |  | Daytime sleepiness |
|  | Sleep-wake problem |  | Daytime sleepiness |
| CBCL | Nightmares |  | Parasomnias |
|  | Overtired |  | Daytime sleepiness |
|  | Sleep less than other kids |  | Sleep duration |
|  | Sleep more than other kids |  | Daytime sleepiness |
|  | Total sleep problem |  | Total sleep problem |
| CSQ | Total score |  | Total sleep problem |

Abbreviations: CBCL=Child Behavior Checklist, CSHQ=the Children's Sleep Habits Questionnaire, CSQ=the Child Sleep Questionnaire, JSQ-P=Japanese Sleep Questionnaire for Preschoolers, PSQ, Pediatric Sleep questionnaire, SDSC=the Sleep Disturbance Scale for Children.

## **eTable 5.2. Unification of parameters for ASD core symptoms**

| Sleep measurements | Original parameters | Used as original | United to |
| --- | --- | --- | --- |
| Aberrant Behavior Checklist | Inappropriate speech |  | Social and communication problem |
|  | Repetitive behavior |  | Restricted and repetitive behavior |
|  | Stereotypic behavior |  | Restricted and repetitive behavior |
| adi-r | Communication verbal total |  | Social and communication problem |
|  | Restricted-Stereotyped behavior total |  | Restricted and repetitive behavior |
|  | Social total |  | Social and communication problem |
|  | Sum of subscales |  | Total core symptoms |
| ados | Social affect severity |  | Social and communication problem |
|  | Restricted and repetitive behavior severity |  | Restricted and repetitive behavior |
|  | Calibrated severity score |  | Total core symptoms |
|  | Total severity |  | Total core symptoms |
| ados-2 | Social affect calibrated severity scores |  | Social and communication problem |
|  | Restricted and repetitive behavior calibrated severity scores |  | Restricted and repetitive behavior |
|  | Total calibrated severity scores |  | Total core symptoms |
| cars | Total score |  | Total core symptoms |
| rbq-2 | Total score |  | Restricted and repetitive behavior |
| rbs-r | Total score |  | Restricted and repetitive behavior |
| Short sensory profile | Total score |  | Restricted and repetitive behavior |
| srs | Autistic mannerism |  | Restricted and repetitive behavior |
|  | Restricted and repetitive behavior |  | Restricted and repetitive behavior |
|  | Total score |  | Social and communication problem |
| srs-2 | Autistic mannerism |  | Restricted and repetitive behavior |
|  | Restricted and repetitive behavior |  | Restricted and repetitive behavior |
|  | Total score |  | Social and communication problem |
| SDQ | Peer problems |  | Social and communication problem |

Abbreviations: ADI-R=Autism Diagnostic Interview-Revised, ADOS=Autism Diagnostic Observation Schedule, CARS=Childhood Autism Rating Scale, RBQ=Repetitive Behaviors Questionnaire, RBS-R=Repetitive Behavior Scales-Revised, SDQ=Strengths and Difficulties Questionnaire, SRS=Social Responsiveness Scale.

## **eTable 5.3. Unification of parameters for behavioral problems**

| Sleep measurements | Original parameters | Used as original | United to |
| --- | --- | --- | --- |
| Cbcl | Withdrawn/Depressed |  | Affective/Anxiety problem |
|  | Somatic complaints | Somatic complaints |  |
|  | Anxious/Depressive | Anxious/Depressive problem |  |
|  | Thought problems | Thought problems |  |
|  | Attention problems | Attention problems |  |
|  | Delinquent |  | Aggressive/Delinquent problem |
|  | Aggressive behavior |  | Aggressive/Delinquent problem |
|  | Externalizing problems | Externalizing problems |  |
|  | Internalizing problems | Internalizing problems |  |
|  | Total behavior problem | Total behavior problem |  |
| cbcl | Affective problems |  | Affective/Anxiety problem |
|  | Somatic complaints |  | Somatic complaints |
|  | Anxiety problems |  | Anxious/Depressive problem |
|  | ADHD problems |  | Attention problems |
|  | Aggressive behavior |  | Aggressive/Delinquent problem |
|  | Externalizing problems |  | Externalizing problems |
| Aberrant Behavior Checklist | Lethargy |  | Affective/Anxiety problem |
|  | Hyperactivity |  | Aggressive/Delinquent problem |
| c-sharp | Physical aggression |  | Aggressive/Delinquent problem |
|  | Hostility |  | Aggressive/Delinquent problem |
| cprs | ADHD index |  | Attention problems |
| sdq | Emotional Symptoms |  | Anxious/Depressive problem |
|  | Hyperactivity/Inattention |  | Attention problems |
|  | Conduct problem |  | Aggressive/Delinquent problem |
| vadprs | Inattention |  | Aggressive/Delinquent problem |
|  | Hyperactivity |  | Aggressive/Delinquent problem |

Abbreviations: CBCL=Child Behavior Checklist; CRPS=Conners Parent Rating Scale, C-SHARP=the Children’s Scale for Hostility and Aggression, SDQ=Strengths and Difficulties Questionnaire, VADPRS=Vanderbit ADHD Diagnostic Parent Rating Scale.

# **eAppendix 6. The list of included studies**

## **eTable 6.1. The list of included studies (Search for correlations between specific sleep problems & ASD core symptoms)**

| **Author, Year** | **Title** |
| --- | --- |
| Gabriels 2005 [1] | Repetitive behaviors in autism: relationships with associated clinical features |
| Gunes 2019 [2] | Sleep problems in children with autism spectrum disorder: clinical correlates and the impact of attention deficit hyperactivity disorder |
| Kang 2020 [3] | Sleep Problems Influence Emotional/Behavioral Symptoms and Repetitive Behavior in Preschool-Aged Children With Autism Spectrum Disorder in the Unique Social Context of China |
| Manelis-Baram 2022 [4] | Sleep Disturbances and Sensory Sensitivities Co-Vary in a Longitudinal Manner in Pre-School Children with Autism Spectrum Disorders |
| Mazurek 2019 [5] | Course and Predictors of Sleep and Co-occurring Problems in Children with Autism Spectrum Disorder |
| Mutluer 2016 [6] | Assessment of sleep problems and related risk factors observed in Turkish children with Autism spectrum disorders |
| Reynolds 2017 [7] | Parent-Reported Problematic Sleep Behaviors in Children with Comorbid Autism Spectrum Disorder and Attention-Deficit/Hyperactivity Disorder |
| Thenhausen 2017 [8] | Sleep problems in adolescents with Asperger syndrome or high-functioning autism |
| Wang 2016 [9] | Sleep Disturbances and Associated Factors in Chinese Children with Autism Spectrum Disorder: A Retrospective and Cross-Sectional Study |
| Wang 2019 [10] | Sensory Processing Problems and Comorbidities in Chinese Preschool Children with Autism Spectrum Disorders |
| Zaidman-Zait 2020 [11] | Factor analysis of the children's sleep habits questionnaire among preschool children with autism spectrum disorder |

1. Gabriels, R.L., et al., Repetitive behaviors in autism: relationships with associated clinical features. Res Dev Disabil, 2005. **26**(2): p. 169-81.

2. Gunes, S., et al., Sleep problems in children with autism spectrum disorder: clinical correlates and the impact of attention deficit hyperactivity disorder. Neuropsychiatr Dis Treat, 2019. **15**: p. 763-771.

3. Kang, Y.-Q., et al., Sleep problems influence emotional/behavioral symptoms and repetitive behavior in preschool-aged children with autism Spectrum disorder in the unique social context of China. Frontiers in psychiatry, 2020. **11**: p. 273.

4. Manelis-Baram, L., et al., Sleep Disturbances and Sensory Sensitivities Co-Vary in a Longitudinal Manner in Pre-School Children with Autism Spectrum Disorders. J Autism Dev Disord, 2022. **52**(2): p. 923-937.

5. Mazurek, M.O., et al., Course and Predictors of Sleep and Co-occurring Problems in Children with Autism Spectrum Disorder. J Autism Dev Disord, 2019. **49**(5): p. 2101-2115.

6. Mutluer, T., S. Karakoc Demirkaya, and O. Abali, Assessment of sleep problems and related risk factors observed in Turkish children with Autism spectrum disorders. Autism Res, 2016. **9**(5): p. 536-42.

7. Reynolds, K.C., et al., Parent-Reported Problematic Sleep Behaviors in Children with Comorbid Autism Spectrum Disorder and Attention-Deficit/Hyperactivity Disorder. Res Autism Spectr Disord, 2017. **39**: p. 20-32.

8. Thenhausen, N., et al., Sleep problems in adolescents with Asperger syndrome or high-functioning autism. Somnologie, 2017. **21**(1): p. 28-36.

9. Wang, G., et al., Sleep Disturbances and Associated Factors in Chinese Children with Autism Spectrum Disorder: A Retrospective and Cross-Sectional Study. Child Psychiatry Hum Dev, 2016. **47**(2): p. 248-58.

10. Wang, G.F., et al., Sensory Processing Problems and Comorbidities in Chinese Preschool Children with Autism Spectrum Disorders. J Autism Dev Disord, 2019. **49**(10): p. 4097-4108.

11. Zaidman-Zait, A., et al., Factor analysis of the children's sleep habits questionnaire among preschool children with autism spectrum disorder. Res Dev Disabil, 2020. **97**: p. 103548.

## **eTable 6.2. The list of included studies (Search for correlations between specific sleep problems & ASD behavioral problems)**

| **Author, Year** | **Title** |
| --- | --- |
| Cremone-Caira 2019 [1] | Relations between caregiver-report of sleep and executive function problems in children with autism spectrum disorder and attention-deficit/hyperactivity disorder |
| Fadini 2015 [2] | Influence of sleep disorders on the behavior of individuals with autism spectrum disorder |
| Hirata 2016 [3] | Sleep problems are more frequent and associated with problematic behaviors in preschoolers with autism spectrum disorder |
| Kang 2020 [4] | Sleep Problems Influence Emotional/Behavioral Symptoms and Repetitive Behavior in Preschool-Aged Children With Autism Spectrum Disorder in the Unique Social Context of China |
| Mazurek 2019 [5] | Course and Predictors of Sleep and Co-occurring Problems in Children with Autism Spectrum Disorder |
| Mazurek 2016 [6] | Sleep and Behavioral Problems in Children with Autism Spectrum Disorder |
| Mutluer 2016 [7] | Assessment of sleep problems and related risk factors observed in Turkish children with Autism spectrum disorders |
| Phung 2019 [8] | Poor sleep quality among adolescents with ASD is associated with depressive symptoms, problem behaviors, and conflicted family relationships |
| Reynolds 2017 [9] | Parent-Reported Problematic Sleep Behaviors in Children with Comorbid Autism Spectrum Disorder and Attention-Deficit/Hyperactivity Disorder |
| Thenhausen 2017 [10] | Sleep problems in adolescents with Asperger syndrome or high-functioning autism |
| Wang 2016 [11] | Sleep Disturbances and Associated Factors in Chinese Children with Autism Spectrum Disorder: A Retrospective and Cross-Sectional Study |
| Zaidman-Zait 2020 [12] | Factor analysis of the children's sleep habits questionnaire among preschool children with autism spectrum disorder |

1. Cremone-Caira, A., et al., Relations between caregiver-report of sleep and executive function problems in children with autism spectrum disorder and attention-deficit/hyperactivity disorder. Res Dev Disabil, 2019. **94**: p. 103464.

2. Fadini, C.C., et al., Influence of sleep disorders on the behavior of individuals with autism spectrum disorder. Front Hum Neurosci, 2015. **9**: p. 347.

3. Hirata, I., et al., Sleep problems are more frequent and associated with problematic behaviors in preschoolers with autism spectrum disorder. Res Dev Disabil, 2016. **49-50**: p. 86-99.

4. Kang, Y.Q., et al., Sleep Problems Influence Emotional/Behavioral Symptoms and Repetitive Behavior in Preschool-Aged Children With Autism Spectrum Disorder in the Unique Social Context of China. Front Psychiatry, 2020. **11**: p. 273.

5. Mazurek, M.O., et al., Course and Predictors of Sleep and Co-occurring Problems in Children with Autism Spectrum Disorder. J Autism Dev Disord, 2019. **49**(5): p. 2101-2115.

6. Mazurek, M.O. and K. Sohl, Sleep and behavioral problems in children with autism spectrum disorder. Journal of autism and developmental disorders, 2016. **46**(6): p. 1906-1915.

7. Mutluer, T., S. Karakoc Demirkaya, and O. Abali, Assessment of sleep problems and related risk factors observed in Turkish children with Autism spectrum disorders. Autism Res, 2016. **9**(5): p. 536-42.

8. Phung, J.N., M.M. Abdullah, and W.A. Goldberg, Poor sleep quality among adolescents with ASD is associated with depressive symptoms, problem behaviors, and conflicted family relationships. Focus on Autism and Other Developmental Disabilities, 2019. **34**(3): p. 173-182.

9. Reynolds, K.C., et al., Parent-Reported Problematic Sleep Behaviors in Children with Comorbid Autism Spectrum Disorder and Attention-Deficit/Hyperactivity Disorder. Res Autism Spectr Disord, 2017. **39**: p. 20-32.

10. Thenhausen, N., et al., Sleep problems in adolescents with Asperger syndrome or high-functioning autism. Somnologie, 2017. **21**(1): p. 28-36.

11. Wang, G., et al., Sleep Disturbances and Associated Factors in Chinese Children with Autism Spectrum Disorder: A Retrospective and Cross-Sectional Study. Child Psychiatry Hum Dev, 2016. **47**(2): p. 248-58.

12. Zaidman-Zait, A., et al., Factor analysis of the children's sleep habits questionnaire among preschool children with autism spectrum disorder. Res Dev Disabil, 2020. **97**: p. 103548.

## **eTable 6.3. The list of included studies (Search for correlations between ASD core symptoms & ASD behavioral problems)**

| **Author, Year** | **Title** |
| --- | --- |
| Factor 2017 [1] | Does the Presence of Anxiety and ADHD Symptoms Add to Social Impairment in Children with Autism Spectrum Disorder? |
| Gabriels 2005 [2] | Repetitive behaviors in autism: relationships with associated clinical features |
| Galligan 2022 [3] | Brief Report: Emotional and Behavioral Problems Among Young Children with ASD: An Exploratory Study of ADOS E-Codes and Child Characteristics |
| Kim 2021 [4] | State and Trait Anxiety of Adolescents with Autism Spectrum Disorders |
| Mazurek 2019 [5] | Course and Predictors of Sleep and Co-occurring Problems in Children with Autism Spectrum Disorder |
| McVey 2018 [6] | Social difficulties in youth with autism with and without anxiety and ADHD symptoms |
| Muskett 2019 [7] | Repetitive behaviors in Autism Spectrum Disorder: Associations with depression and anxiety symptoms |
| Saito 2017 [8] | Association Between Autistic Traits in Preschool Children and Later Emotional/Behavioral Outcomes |
| Wang 2019 [9] | Sensory Processing Problems and Comorbidities in Chinese Preschool Children with Autism Spectrum Disorders |
| Zaidman-Zait 2020 [10] | Factor analysis of the children's sleep habits questionnaire among preschool children with autism spectrum disorder |

1. Factor, R.S., et al., Does the Presence of Anxiety and ADHD Symptoms Add to Social Impairment in Children with Autism Spectrum Disorder? J Autism Dev Disord, 2017. **47**(4): p. 1122-1134.

2. Gabriels, R.L., et al., Repetitive behaviors in autism: relationships with associated clinical features. Res Dev Disabil, 2005. **26**(2): p. 169-81.

3. Galligan, M.L., et al., Brief Report: Emotional and Behavioral Problems Among Young Children with ASD: An Exploratory Study of ADOS E-Codes and Child Characteristics. J Autism Dev Disord, 2022. **52**(10): p. 4597-4604.

4. Kim, S.Y., et al., State and Trait Anxiety of Adolescents with Autism Spectrum Disorders. Psychiatry Investig, 2021. **18**(3): p. 257-265.

5. Mazurek, M.O., et al., Course and Predictors of Sleep and Co-occurring Problems in Children with Autism Spectrum Disorder. J Autism Dev Disord, 2019. **49**(5): p. 2101-2115.

6. McVey, A.J., et al., Social difficulties in youth with autism with and without anxiety and ADHD symptoms. Autism Res, 2018. **11**(12): p. 1679-1689.

7. Muskett, A., et al., Repetitive behaviors in Autism Spectrum Disorder: Associations with depression and anxiety symptoms. Research in Autism Spectrum Disorders, 2019. **68**: p. 101449.

8. Saito, A., et al., Association Between Autistic Traits in Preschool Children and Later Emotional/Behavioral Outcomes. J Autism Dev Disord, 2017. **47**(11): p. 3333-3346.

9. Wang, G.F., et al., Sensory Processing Problems and Comorbidities in Chinese Preschool Children with Autism Spectrum Disorders. J Autism Dev Disord, 2019. **49**(10): p. 4097-4108.

10. Zaidman-Zait, A., et al., Factor analysis of the children's sleep habits questionnaire among preschool children with autism spectrum disorder. Res Dev Disabil, 2020. **97**: p. 103548.

# **eAppendix 7. Result of the study quality assessment (AXIS score)**

## **eTable 7.1. Result of the AXIS score**

|  | **Author, year** | | | | | | | | | | | | | | | | | | | | | | |
| --- | --- | --- | --- | --- | --- | --- | --- | --- | --- | --- | --- | --- | --- | --- | --- | --- | --- | --- | --- | --- | --- | --- | --- |
|  | Cremone –Caira 2019 | Factor 2017 | Fadini 2015 | Gabriels 2005 | Galligan 2021 | Gunes 2019 | Hirata 2016 | Kang 2020 | Kim 2021 | Manelis-Baram 2022 | Mazurek 2016 | Mazurek 2019 | McVey 2018 | Muskett 2019 | Mutluer 2016 | Phung 2018 | Reynolds 2017 | Saito 2017 | Thenhausen 2017 | Wang 2016 | Wang 2019 | Zaidman-Zait 2020 |  |
| **Introduction** | | | | | | | | | | | | | | | | | | | | | | | |
| Were the aims/objectives of the study clear? | * | * | * | * | * | * | * | * | * | * | * | * | * | * | * | * | * | * | * | * | * | * |  |
| **Methods** | | | | | | | | | | | | | | | | | | | | | | | |
| Was the study design appropriate for the stated aim(s)? | No | * | * | * | * | * | * | * | * | * | * | * | * | * | * | * | * | * | * | * | * | * |  |
| Was the sample size justified? | * | * | * | No | * | * | * | * | * | * | * | * | * | * | * | * | * | * | No | * | * | * |  |
| Was the target/reference population clearly defined? (Is it clear who the research was about?) | No | * | * | * | * | * | * | * | * | * | * | * | * | * | * | * | * | * | * | * | * | * |  |
| Was the sample frame taken from an appropriate population base so that it closely represented the target/reference population under investigation? | * | * | * | No | * | * | * | * | * | * | * | * | * | * | * | * | * | * | * | * | * | * |  |
| Was the selection process likely to select subjects/participants that were representative of the target/reference population under investigation? | No | * | No | * | No | * | * | * | * | * | * | * | * | * | * | * | * | * | No | * | * | * |  |
| Were measures undertaken to address and categorise non-responders? | * | * | * | * | * | * | * | * | * | * | * | * | * | * | * | * | * | * | * | * | * | * |  |
| Were the risk factor and outcome variables measured appropriate to the aims of the study? | * | * | * | * | * | * | * | * | * | * | * | * | * | * | * | * | * | * | * | * | * | * |  |
| Were the risk factor and outcome variables measured correctly using instruments/measurements that had been trialed, piloted or published previously? | * | * | * | * | * | * | * | * | * | * | * | * | * | * | * | * | * | * | * | * | * | * |  |
| Is it clear what was used to determined statistical significance and/or precision estimates? (eg, p values, CIs) | * | * | * | * | * | * | * | * | * | * | * | * | * | * | * | * | * | * | * | * | * | * |  |
| Were the methods (including statistical methods) sufficiently described to enable them to be repeated? | * | * | * | * | * | * | * | * | * | * | * | * | * | * | * | * | * | * | * | * | * | * |  |
| **Results** | | | | | | | | | | | | | | | | | | | | | | | |
| Were the basic data adequately described? | * | * | * | * | * | * | * | * | * | * | * | * | * | * | * | * | * | * | * | * | * | * |  |
| Does the response rate raise concerns about non-response bias? | * | Yes | * | * | * | * | * | * | * | Yes | * | Yes | * | * | * |  | Yes | Yes | * | * | * | * |  |
| If appropriate, was information about non-responders described? |  | * |  |  |  |  |  |  |  | * |  | * |  |  |  | * | * | * |  |  |  |  |  |
| Were the results internally consistent? | * | * | * | * | * | * | * | * | * | * | * | * | * | * | * | * | * | * | * | * | * | * |  |
| Were the results for the analyses described in the methods, presented? | * | * | * | * | * | * | * | * | * | * | * | * | * | * | * | * | * | * | * | * | * | * |  |
| **Discussion** | | | | | | | | | | | | | | | | | | | | | | | |
| Were the authors’ discussions and conclusions justified by the results? | * | * | * | * | * | * | * | * | * | * | * | * | * | * | * | * | * | * | * | * | * | * |  |
| Were the limitations of the study discussed? | * | * | No | * | * | * | * | * | * | * | * | * | * | * | * | * | * | * | * | * | * | * |  |
| **Other** | | | | | | | | | | | | | | | | | | | | | | | |
| Were there any funding sources or conflicts of interest that may affect the authors’ interpretation of the results? | * | * | * | * | * | * | * | * | * | * | * | * | * | * | * | * | Yes | * | * | * | * | * |  |
| Was ethical approval or consent of participants attained? | * | * | * | * | * | * | * | * | * | * | * | * | * | * | * | * | * | * | * | * | * | * |  |
| **Total score** | **17** | **20** | **18** | **18** | **19** | **20** | **20** | **20** | **20** | **20** | **20** | **20** | **20** | **20** | **20** | **17** | **19** | **20** | **18** | **20** | **20** | **20** |  |

Abbreviations: AXIS= the Appraisal tool for Cross-Sectional Studies

# **eAppendix 8. Pooled correlations between sleep problems & ASD core symptoms**

## **eTable 8.1. Pooled correlations between sleep problems & ASD core symptoms**

|  | **Social communication problem** | **Restricted and repetitive behavior** | **Total core symptoms** |
| --- | --- | --- | --- |
| **Bedtime resistance** | 0.0294 | 0.2555 | 0.1242 |
| **Daytime sleepiness** | 0.0111 | 0.1271 | 0.0633 |
| **Night waking** | 0.0642 | 0.1119 | 0.1599 |
| **Parasomnias** | 0.0226 | 0.0693 | 0.1208 |
| **Sleep anxiety** | 0.0909 | **0.289** | 0.2597 |
| **Sleep duration** | 0.0637 | -0.0752 | -0.0252 |
| **Sleep disordered breathing** | -0.0243 | 0.0486 | 0.0136 |
| **Sleep onset delay** | 0.233 | **0.192** | 0.0969 |
| **Total sleep problem** | 0.1441 | 0.1941 | 0.2931 |

Statistically significant correlations were highlighted in bold.

## **eTable 8.2. Statistical results of meta-analyses between sleep problems & ASD core symptoms**

| **Meta-analysis** | | | | | | | **Heterogeneity** | | | **Eggers' test** | | **Moderators** |
| --- | --- | --- | --- | --- | --- | --- | --- | --- | --- | --- | --- | --- |
| **Sleep parameters** | **Core symptoms of ASD** | **k** | **N_ASD** | **Correlation (95% CI)** | **t (or z)** | **p-values** | **Q** | **p-values** | ***I^2^*** | **t** | **p-values** |  |
| **Bedtime resistance** | Social communication problem | 3 | 593 | 0.0294 (-0.1183 to 0.1759) | 0.85 | 0.4831 | 1.39 | 0.4995 | 0 | 0.767 | 0.5833 |  |
|  | Restricted and repetitive behavior | 2 | 533 | 0.2555 (-0.9802 to 0.9930) | 1.3 | 0.4186 | 21.39 | <0.0001 | 95.3 | NA | NA | Medication use status |
|  | Total core symptoms | 3 | 645 | 0.1242 (-0.1671 to 0.3956) | 1.83 | 0.2087 | 6.21 | 0.0448 | 67.8 | 0.598 | 0.657 | Medication use status |
| **Daytime sleepiness** | Social communication problem | 5 | 693 | 0.0111 (-0.1429 to 0.1646) | 0.2 | 0.8518 | 6.35 | 0.1742 | 37 | 2.255 | 0.1095 | Medication use status |
|  | Restricted and repetitive behavior | 3 | 618 | 0.1271 (-0.3256 to 0.5323) | 1.18 | 0.3592 | 9.69 | 0.0079 | 79.4 | 2.098 | 0.2832 |  |
|  | Total core symptoms | 4 | 730 | 0.0633 (-0.1206 to 0.2429) | 1.09 | 0.3543 | 7.88 | 0.0486 | 61.9 | 0.084 | 0.9409 |  |
| **Night waking** | Social communication problem | 3 | 593 | 0.0642 (-0.2458 to 0.3624) | 0.88 | 0.4727 | 7.04 | 0.0296 | 71.6 | -0.029 | 0.9818 | Medication use status |
|  | Restricted and repetitive behavior | 2 | 533 | 0.1119 (-0.5201 to 0.6648) | 2.07 | 0.2861 | 1.55 | 0.2137 | 35.3 | NA | NA |  |
|  | Total core symptoms | 3 | 645 | 0.1599 (-0.2386 to 0.5123) | 1.72 | 0.2284 | 13.01 | 0.0015 | 84.6 | 0.307 | 0.8103 | Medication use status |
| **Parasomnias** | Social communication problem | 4 | 412 | 0.0226 (-0.1302 to 0.1743) | 0.47 | 0.672 | 2.79 | 0.4247 | 0 | 0.255 | 0.8223 |  |
|  | Restricted and repetitive behavior | 2 | 337 | 0.0693 (-0.9818 to 0.9862) | 0.36 | 0.7772 | 8.94 | 0.0028 | 88.8 | NA | NA |  |
|  | Total core symptoms | 3 | 449 | 0.1208 (-0.1691 to 0.3914) | 1.79 | 0.2157 | 3.36 | 0.1866 | 40.4 | -0.92 | 0.5265 | Medication use status |
| **Sleep anxiety** | Social communication problem | 2 | 312 | 0.0909 (-0.0977 to 0.2733) | 6.12 | 0.103 | 0.07 | 0.7945 | 0 | NA | NA |  |
|  | Restricted and repetitive behavior | 1 | 252 | 0.2890 (0.1716 to 0.3983) | 4.69 | <0.0001 |  |  |  |  |  |  |
|  | Total core symptoms | 2 | 364 | 0.2597 (-0.9649 to 0.9877) | 1.48 | 0.3778 | 9.76 | 0.0018 | 89.8 | NA | NA |  |
| **Sleep duration** | Social communication problem | 4 | 480 | 0.0637 (-0.2660 to 0.3801) | 0.6 | 0.5887 | 10.8 | 0.0129 | 72.2 | 0.939 | 0.4468 |  |
|  | Restricted and repetitive behavior | 3 | 420 | -0.0752 (-0.4609 to 0.3343) | -0.77 | 0.5235 | 7.14 | 0.0281 | 72 | -2.109 | 0.2818 | Medication use status |
|  | Total core symptoms | 3 | 420 | -0.0252 (-0.5462 to 0.5098) | -0.18 | 0.8704 | 15.55 | 0.0004 | 87.1 | -8.183 | 0.0774 | Medication use status |
| **Sleep disordered breathing** | Social communication problem | 4 | 608 | -0.0243 (-0.0717 to 0.0231) | -1.63 | 0.2012 | 0.4 | 0.9407 | 0 | -1.036 | 0.4092 |  |
|  | Restricted and repetitive behavior | 2 | 533 | 0.0486 (-0.9963 to 0.9970) | 0.19 | 0.8786 | 33.3 | <0.0001 | 97 | NA | NA | Medication use status |
|  | Total core symptoms | 3 | 645 | 0.0136 (-0.4073 to 0.4298) | 0.13 | 0.9076 | 14.23 | 0.0008 | 85.9 | -0.278 | 0.8276 |  |
| **Sleep onset delay** | Social communication problem | 2 | 312 | 0.2330 (-0.7931 to 0.9145) | 2.29 | 0.2621 | 2.17 | 0.1403 | 54 | NA | NA |  |
|  | Restricted and repetitive behavior | 1 | 252 | 0.1920 (0.0701 to 0.3083) | 3.07 | 0.0022 |  |  |  |  |  |  |
|  | Total core symptoms | 2 | 364 | 0.0969 (-0.9947 to 0.9964) | 0.4 | 0.7559 | 17.6 | <0.0001 | 94.3 | NA | NA |  |
| **Total sleep problem** | Social/Communication | 6 | 559 | 0.1441 (-0.1179 to 0.3873) | 1.42 | 0.2161 | 16.59 | 0.0053 | 69.9 | 1.5 | 0.2079 |  |
|  | Restricted and repetitive behavior | 8 | 1016 | 0.1941 (-0.1116 to 0.4663) | 1.51 | 0.1758 | 61.73 | <0.0001 | 88.7 | -0.389 | 0.711 |  |
|  | Total core symptoms | 4 | 532 | 0.0992 (-0.2807 to 0.4522) | 0.82 | 0.474 | 25.98 | <0.0001 | 88.5 | -12.8 | 0.006 | Medication use status |

Abbreviations: ASD=autism spectrum disorder, CI=confidence interval, N=the number of participants, NA=not available, k=the number of studies.

## **eTable 8.3. Effect size correction using the trim-and-fill method (Total sleep problem & Total core symptoms)**

| **Meta-analysis** | | | | | **Heterogeneity** | | |
| --- | --- | --- | --- | --- | --- | --- | --- |
|  | **k** | **Correlation (95% CI)** | **t** | **p-values** | **Q** | **p-values** | ***I^2^*** |
| **Before correction** | 4 | 0.0992 (-0.2807 to 0.4522) | 0.82 | 0.474 | 25.98 | <0.0001 | 88.5 |
| **After correction** | 6 | 0.2931 (-0.0949 to 0.6038) | 1.95 | 0.108 | 61.49 | <0.0001 | 91.9 |

* Two studies were added for correction

Abbreviations: CI=confidence interval, k=the number of studies.

# **eAppendix 9. Pooled correlations between sleep problems & behavioral problems**

## **eTable 9.1. Pooled correlations between sleep problems & behavioral problems**

|  | **Affective/Anxiety problem** | **Aggressive/Delinquent problem** | **Attention problem** | **Somatic complaints** | **Thought problems** | **Internalizing problems** | **Externalizing problems** | **Total behavior problem** |
| --- | --- | --- | --- | --- | --- | --- | --- | --- |
| **Bedtime resistance** | **0.214** | **0.1839** | 0.1912 | NA | NA | NA | NA | 0.2369 |
| **Daytime sleepiness** | **0.2954** | **0.1707** | **0.13** | 0 | **0.45** | 0.3086 | 0.2316 | **0.2871** |
| **Night waking** | **0.1931** | **0.2524** | **0.2118** | NA | NA | NA | NA | 0.2441 |
| **Parasomnias** | **0.1973** | 0.1664 | **0.2411** | 0.28 | **0.42** | 0.2219 | 0.2001 | **0.2416** |
| **Sleep anxiety** | 0.2203 | 0.104 | 0.2863 | NA | NA | NA | NA | 0.3384 |
| **Sleep duration** | 0.2132 | 0.175 | 0.1349 | NA | NA | NA | NA | 0.26 |
| **Sleep disordered breathing** | 0.1789 | 0.2123 | **0.196** | 0 | NA | 0.2547 | 0.3746 | 0.2711 |
| **Sleep onset delay** | 0.1673 | **0.1848** | 0.1888 | NA | NA | NA | NA | **0.339** |
| **Total sleep problem** | **0.453** | **0.409** | **0.3342** | **0.4004** | NA | **0.3759** | **0.4048** | **0.4294** |

Statistically significant correlations were highlighted in bold.

Abbreviations: NA=not available.

## **eTable 9.2. Statistical results of meta-analyses between sleep problems & behavioral problems**

| **Meta-analysis** | | | | | | | **Heterogeneity** | | | **Eggers' test** | | **Moderators** |
| --- | --- | --- | --- | --- | --- | --- | --- | --- | --- | --- | --- | --- |
| **Sleep parameters** | **Behavioral parameters** | **k** | **N_ASD** | **Correlation (95% CI)** | **t (or z)** | **p-values** | **Q** | **p-values** | ***I^2^*** | **t** | **p-values** |  |
| Bedtime resistance | Affective/Anxiety problem | 3 | 593 | 0.2140 (0.1067 to 0.3164) | 8.48 | 0.0136 | 0.77 | 0.6813 | 0 | -0.427 | 0.7432 |  |
|  | Aggressive/Delinquent problem | 4 | 674 | 0.1839 (0.1141to 0.2520) | 8.29 | 0.0037 | 1 | 0.801 | 0 | 0.627 | 0.595 |  |
|  | Attention problem | 4 | 674 | 0.1912 (-0.0694 to 0.4274) | 2.34 | 0.1011 | 8 | 0.046 | 62.5 | 0.2 | 0.86 |  |
|  | Total behavior problem | 2 | 312 | 0.2369 (-0.5949 to 0.8237) | 3.31 | 0.1867 | 1.29 | 0.2558 | 22.6 | NA | NA |  |
| Daytime sleepiness | Affective/Anxiety problem | 5 | 636 | 0.2954 (0.2182 to 0.3689) | 10.22 | 0.0005 | 2.2 | 0.6982 | 0 | -1.676 | 0.1923 |  |
|  | Aggressive/Delinquent problem | 5 | 689 | 0.1707 (0.0469 to 0.2893) | 3.82 | 0.0188 | 5.12 | 0.2755 | 21.8 | 0.668 | 0.5518 |  |
|  | Attention problem | 6 | 774 | 0.1300 (0.0529 to 0.2055) | 4.32 | 0.0076 | 3.46 | 0.6298 | 0 | 0.371 | 0.7294 |  |
|  | Somatic complaints | 1 | 15 | 0.0000 (-0.5123 to 0.5123) | 0 | 1 |  |  |  |  |  |  |
|  | Thought problems | 1 | 45 | 0.4500 (0.1803 to 0.6568) | 3.14 | 0.0017 |  |  |  |  |  |  |
|  | Internalizing problems | 3 | 293 | 0.3086 (-0.0555 to 0.6002) | 3.66 | 0.0671 | 3.11 | 0.2111 | 35.7 | -0.19 | 0.8805 |  |
|  | Externalizing problems | 3 | 236 | 0.2316 (-0.0866 to 0.5069) | 3.15 | 0.088 | 2.37 | 0.3065 | 15.4 | 0.207 | 0.8699 |  |
|  | Total behavior problem | 5 | 565 | 0.2871 (0.1189 to 0.4393) | 4.66 | 0.0096 | 8.91 | 0.0635 | 55.1 | 0.72 | 0.5234 | Medication use status |
| Night waking | Affective/Anxiety problem | 3 | 593 | 0.1931 (0.0144 to 0.3597) | 4.65 | 0.0433 | 2.01 | 0.3659 | 0.5 | 0.512 | 0.6986 |  |
|  | Aggressive/Delinquent problem | 4 | 674 | 0.2524 (0.0600 to 0.4268) | 4.15 | 0.0255 | 6.95 | 0.0734 | 56.8 | 0.609 | 0.6043 | Medication, Percentage of boys |
|  | Attention problem | 4 | 674 | 0.2118 (0.0547 to 0.3587) | 4.27 | 0.0236 | 4.32 | 0.2294 | 30.5 | 1.351 | 0.3093 |  |
|  | Total behavior problem | 2 | 312 | 0.2441 (-0.1178 to 0.5487) | 8.61 | 0.0736 | 0.26 | 0.613 | 0 | NA | NA |  |
| Parasomnias | Affective/Anxiety problem | 3 | 327 | 0.1973 (0.0624 to 0.3251) | 6.26 | 0.0246 | 0.65 | 0.723 | 0 | -0.343 | 0.7894 |  |
|  | Aggressive/Delinquent problem | 4 | 408 | 0.1664 (-0.1714 to 0.4692) | 1.57 | 0.2151 | 12.93 | 0.0048 | 768 | 0.752 | 0.5306 | Medication use status |
|  | Attention problem | 5 | 493 | 0.2411 (0.1852 to 0.2955) | 11.65 | 0.0003 | 0.85 | 0.9313 | 0 | 0.063 | 0.9535 |  |
|  | Somatic complaints | 1 | 15 | 0.2800 (-0.2712 to 0.6929) | 1 | 0.319 |  |  |  |  |  |  |
|  | Thought problems | 1 | 45 | 0.4200 (0.1442 to 0.6352) | 2.9 | 0.0037 |  |  |  |  |  |  |
|  | Internalizing problems | 3 | 293 | 0.2219 (-0.0288 to 0.4464) | 3.82 | 0.0624 | 1.8 | 0.4067 | 0 | 0.13 | 0.9177 |  |
|  | Externalizing problems | 2 | 208 | 0.2001 (-0.3070 to 0.6187) | 4.96 | 0.1267 | 0.34 | 0.5608 | 0 | NA | NA |  |
|  | Total behavior problem | 4 | 520 | 0.2416 (0.1347 to 0.3429) | 7.07 | 0.0058 | 1.85 | 0.6036 | 0 | 0.15 | 0.8948 |  |
| Sleep anxiety | Affective/Anxiety problem | 2 | 312 | 0.2203 (-0.7085 to 0.8698) | 2.57 | 0.2364 | 1.65 | 0.199 | 39.4 | NA | NA |  |
|  | Aggressive/Delinquent problem | 3 | 393 | 0.2106 (-0.1079 to 0.4899) | 2.86 | 0.1039 | 3.84 | 0.1469 | 47.9 | 26.041 | 0.0244 | Medication use status |
|  | Attention problem | 3 | 393 | 0.2863 (-0.3917 to 0.7628) | 1.79 | 0.2155 | 15.23 | 0.0005 | 86.9 | -0.605 | 0.6536 |  |
|  | Total behavior problem | 2 | 312 | 0.3384 (-0.3829 to 0.8033) | 5.92 | 0.1065 | 1.04 | 0.3087 | 3.5 | NA | NA |  |
| Sleep duration | Affective/Anxiety problem | 2 | 312 | 0.2132 (-0.0562 to 0.4537) | 10.09 | 0.0629 | 0.14 | 0.7072 | 0 | NA | NA |  |
|  | Aggressive/Delinquent problem | 3 | 393 | 0.2581 (-0.0161 to 0.4961) | 4.06 | 0.0558 | 2.9 | 0.234 | 31.1 | 120.294 | 0.0053 | Medication use status |
|  | Attention problem | 4 | 478 | 0.1349 (-0.1646 to 0.4117) | 1.43 | 0.2478 | 11.86 | 0.0079 | 74.7 | 3.388 | 0.0772 | Medication use status |
|  | Total behavior problem | 2 | 312 | 0.2600 (-0.9420 to 0.9796) | 1.67 | 0.3431 | 4.78 | 0.0288 | 79.1 | NA | NA | Medication use status |
| Sleep disordered breathing | Affective/Anxiety problem | 5 | 672 | 0.1789 (-0.0663 to 0.4037) | 2.03 | 0.1121 | 13.21 | 0.0103 | 69.7 | 0.725 | 0.5208 |  |
|  | Aggressive/Delinquent problem | 6 | 753 | 0.2123 (-0.1453 to 0.5208) | 1.53 | 0.1863 | 42.95 | <0.0001 | 88.4 | 1.634 | 0.1776 |  |
|  | Attention problem | 6 | 753 | 0.1960 (0.1065 to 0.2823) | 5.57 | 0.0026 | 4.67 | 0.4574 | 0 | 1.754 | 0.1544 |  |
|  | Somatic complaints | 1 | 15 | 0.0000 (-0.5123 to 0.5123) | 0 | 1 |  |  |  |  |  |  |
|  | Internalizing problems | 3 | 272 | 0.2547 (-0.2604 to 0.6569) | 2.13 | 0.1673 | 5.97 | 0.0504 | 66.5 | 0.386 | 0.7655 | Medication use status |
|  | Externalizing problems | 3 | 272 | 0.3746 (-0.2693 to 0.7870) | 2.53 | 0.1272 | 8.9 | 0.0117 | 77.5 | 1.617 | 0.3526 |  |
|  | Total behavior problem | 5 | 584 | 0.2711 (-0.0309 to 0.5278) | 2.5 | 0.0669 | 18.66 | 0.0009 | 78.6 | 1.652 | 0.197 |  |
| Sleep onset delay | Affective/Anxiety problem | 2 | 312 | 0.1673 (-0.8129 to 0.9002) | 1.65 | 0.3477 | 2.14 | 0.1436 | 53.3 | NA | NA |  |
|  | Aggressive/Delinquent problem | 3 | 393 | 0.1848 (0.0019 to 0.3558) | 4.35 | 0.0491 | 1.42 | 0.4914 | 0 | -1.033 | 0.4898 |  |
|  | Attention problem | 3 | 393 | 0.1888 (-0.0010 to 0.3655) | 4.28 | 0.0505 | 1.53 | 0.465 | 0 | -0.075 | 0.9523 |  |
|  | Total behavior problem | 2 | 312 | 0.3390 (0.0221 to 0.5940) | 13.56 | 0.0469 | 0.21 | 0.6488 | 0 | NA | NA |  |
| Total sleep problems | Affective/Anxiety problem | 6 | 828 | 0.4530 (0.3356 to 0.5566) | 9.01 | 0.0003 | 9.04 | 0.1073 | 44.7 | -0.757 | 0.4913 |  |
|  | Aggressive/Delinquent problem | 6 | 828 | 0.4090 (0.2112 to 0.5747) | 5.08 | 0.0039 | 22.92 | 0.0003 | 78.2 | 1.237 | 0.2838 |  |
|  | Attention problem | 8 | 1014 | 0.3342 (0.2613 to 0.4034) | 10.26 | <0.0001 | 7.93 | 0.3385 | 11.8 | 0.058 | 0.9554 |  |
|  | Somatic complaints | 3 | 452 | 0.4004 (0.2693 to 0.5169) | 12.33 | 0.0065 | 1.05 | 0.5922 | 0 | -0.94 | 0.5197 |  |
|  | Internalizing problems | 4 | 357 | 0.3759 (0.1971 to 0.5305) | 6.43 | 0.0076 | 3.48 | 0.3228 | 13.9 | 0.018 | 0.987 |  |
|  | Externalizing problems | 3 | 272 | 0.4048 (0.0091 to 0.6909) | 4.4 | 0.0481 | 3.63 | 0.1628 | 44.9 | 0.252 | 0.843 | Medication use status |
|  | Total behavior problem | 5 | 584 | 0.4294 (0.2994 to 0.5438) | 8.48 | 0.0011 | 5.92 | 0.205 | 32.5 | 0.941 | 0.4163 |  |

Abbreviations: ASD=autism spectrum disorder, CI=confidence interval, N=the number of participants, NA=not available, k=the number of studies.

## **eTable 9.3. Effect size correction using the trim-and-fill method (Sleep anxiety & Aggressive/Delinquent problem)**

| **Meta-analysis** | | | | | **Heterogeneity** | | |
| --- | --- | --- | --- | --- | --- | --- | --- |
|  | **k** | **Correlation (95% CI)** | **t** | **p-values** | **Q** | **p-values** | ***I^2^*** |
| **Before correction** | 3 | 0.2106 (-0.1079 to 0.4899) | 2.86 | 0.1039 | 3.84 | 0.1469 | 47.9 |
| **After correction** | 5 | 0.1040 (-0.1476 to 0.3430) | 1.15 | 0.316 | 11.72 | 0.0195 | 65.9 |

* Two studies were added for correction

Abbreviations: CI=confidence interval, k=the number of studies.

## **eTable 9.4. Effect size correction using the trim-and-fill method (Sleep duration & Aggressive/Delinquent problem)**

| **Meta-analysis** | | | | | **Heterogeneity** | | |
| --- | --- | --- | --- | --- | --- | --- | --- |
|  | **k** | **Correlation (95% CI)** | **t** | **p-values** | **Q** | **p-values** | ***I^2^*** |
| **Before correction** | 3 | 0.2581 (-0.0161 to 0.4961) | 4.06 | 0.0558 | 2.9 | 0.234 | 31.1 |
| **After correction** | 5 | 0.1750 (-0.0384 to 0.3732) | 2.28 | 0.0848 | 8.84 | 0.0651 | 54.8 |

* Two studies were added for correction

Abbreviations: CI=confidence interval, k=the number of studies.

# **eAppendix 10. Pooled correlations between ASD core symptoms & behavioral problems**

## **eTable 10.1. Pooled correlations between ASD core symptoms & behavioral problems**

|  | **Social communication problem** | **Restricted and repetitive behavior** | **Total core symptoms** |
| --- | --- | --- | --- |
| **Affective/Anxiety problem** | 0.3258 | **0.2559** | -0.08 |
| **Aggressive/Delinquent problem** | 0.1314 | 0.2958 | **-0.13** |
| **Attention problem** | 0.3054 | **0.2709** | 0 |
| **Somatic complaints** | NA | 0.1463 | NA |
| **Total behavioral problem** | **0.67** | **-0.313** | -0.05 |

Statistically significant correlations were highlighted in bold.

Abbreviations: NA, not available

## **eTable 10.2. Statistical results of meta-analyses between ASD core symptoms & behavioral problems**

| **Meta-analysis** | | | | | | | **Heterogeneity** | | | **Eggers' test** | | **Moderators** | |
| --- | --- | --- | --- | --- | --- | --- | --- | --- | --- | --- | --- | --- | --- |
| **Behavioral problems** | **Core symptoms** | **k** | **N_ASD** | **Correlation (95% CI)** | **t (or z)** | **p-values** | **Q** | **p-values** | ***I^2^*** | **t** | **p-values** |  |  |
| **Affective/Anxiety problem** | **Social and communication problem** | 4 | 640 | 0.3258 (-0.2023 to 0.7071) | 1.98 | 0.1419 | 72.94 | <0.0001 | 95.9 | 1.065 | 0.398 |  |  |
|  | **Restricted and repetitive behavior** | 9 | 1222 | 0.3826 (0.2217 to 0.5232) | 5.23 | 0.0008 | 51.38 | <0.0001 | 84.4 | 2.603 | **0.035** |  |  |
|  | **Total core symptoms** | 1 | 281 | -0.0800 (-0.1952 to 0.0374) | -1.34 | 0.1813 |  |  |  |  |  |  |  |
| **Aggressive/Delinquent problem** | **Social and communication problem** | 2 | 470 | 0.1314 (-0.9960 to 0.9976) | 0.52 | 0.6951 | 28.87 | <0.0001 | 96.5 | NA | NA |  |  |
|  | **Restricted and repetitive behavior** | 5 | 921 | 0.2958 (-0.0960 to 0.6083) | 2.11 | 0.1025 | 41.8 | <0.0001 | 90.4 | 1.326 | 0.277 |  |  |
|  | **Total core symptoms** | 1 | 281 | -0.1300 (-0.2433 to -0.0132) | -0.218 | 0.0293 |  |  |  |  |  |  |  |
| **Attention problem** | **Social and communication problem** | 4 | 640 | 0.3054 (-0.0834 to 0.6135) | 2.52 | 0.0865 | 40.3 | <0.0001 | 92.6 | 0.624 | 0.596 |  |  |
|  | **Restricted and repetitive behavior** | 6 | 1077 | 0.2709 (0.0550 to 0.4627) | 3.21 | 0.0238 | 41.01 | <0.0001 | 87.8 | 0.415 | 0.699 |  |  |
|  | **Total core symptoms** | 1 | 281 | 0.0000 (-0.1170 to 0.1170) | 0 | 1 |  |  |  |  |  |  |  |
| **Somatic complaints** | **Social and communication problem** | 2 | 437 | 0.1463 (-0.7919 to 0.8789) | 1.53 | 0.3686 | 3.78 | 0.052 | 73.5 | NA | NA |  |  |
| **Total behavioral problem** | **Social and communication problem** | 1 | 233 | 0.6700 (0.5925 to 0.7352) | 12.3 | <0.0001 |  |  |  |  |  |  |  |
|  | **Restricted and repetitive behavior** | 1 | 81 | -0.3130 (-0.4974 to -0.1016) | -2.86 | 0.0042 |  |  |  |  |  |  |  |
|  | **Total core symptoms** | 1 | 233 | -0.0500 (-0.1774 to 0.0790) | -0.76 | 0.4479 |  |  |  |  |  |  |  |

Abbreviations: ASD=autism spectrum disorder, CI=confidence interval, N=the number of participants, NA=not available, k=the number of studies.

## **eTable 10.3. Effect size correction using the trim-and-fill method (Affective/Anxiety problem & Restricted and repetitive behavior)**

| **Meta-analysis** | | | | | **Heterogeneity** | | |
| --- | --- | --- | --- | --- | --- | --- | --- |
|  | **k** | **Correlation (95% CI)** | **t** | **p-values** | **Q** | **p-values** | ***I^2^*** |
| **Before correction** | 9 | 0.3826 (0.2217 to 0.5232) | 5.23 | 0.0008 | 51.38 | <0.0001 | 84.4 |
| **After correction** | 13 | 0.2559 (0.0583 to 0.4342) | 2.8 | 0.0159 | 84.97 | <0.0001 | 85.9 |

* Four studies were added for correction

Abbreviations: CI=confidence interval, k=the number of studies.

# **eAppendix 11. Statistical results of meta-regression (mean age of participants, percentage of boys, mean IQ, and AXIS score****)**

## **eTable 11.1. Statistical results of meta-regression – sleep problems & ASD core symptoms**

| **Sleep parameters** | **Core symptoms** | **Meta-regression moderators** | **k** | **Coefficient (95% CI)** | **p value** | **NA reason** |
| --- | --- | --- | --- | --- | --- | --- |
| **Bedtime resistance** | **Social communication problem** | Mean age of participants |  |  |  | k<4 |
|  |  | Percentage of boys |  |  |  | k<4 |
|  |  | Mean IQ |  |  |  | k<4 |
|  |  | AXIS score |  |  |  | k<4 |
|  | **Restricted and repetitive behavior** | Mean age of participants |  |  |  | k<4 |
|  |  | Percentage of boys |  |  |  | k<4 |
|  |  | Mean IQ |  |  |  | k<4 |
|  |  | AXIS score |  |  |  | k<4 |
|  | **Total core symptoms** | Mean age of participants |  |  |  | k<4 |
|  |  | Percentage of boys |  |  |  | k<4 |
|  |  | Mean IQ |  |  |  | k<4 |
|  |  | AXIS score |  |  |  | k<4 |
| **Daytime sleepiness** | **Social communication problem** | Mean age of participants | 5 | 0.0330 (-0.0018 to 0.0677) | 0.0568 |  |
|  |  | Percentage of boys | 5 | 0.0009 (-0.0588 to 0.0607) | 0.9639 |  |
|  |  | Mean IQ |  |  |  | k<4 |
|  |  | AXIS score | 5 | -2.4915 (-9.3807 to 4.3977) | 0.3332 |  |
|  | **Restricted and repetitive behavior** | Mean age of participants |  |  |  | k<4 |
|  |  | Percentage of boys |  |  |  | k<4 |
|  |  | Mean IQ |  |  |  | k<4 |
|  |  | AXIS score |  |  |  | k<4 |
|  | **Total core symptoms** | Mean age of participants | 4 | 0.0061 (-0.1462 to 0.1584) | 0.8795 |  |
|  |  | Percentage of boys | 4 | -0.0019 (-0.0647 to 0.0609) | 0.9095 |  |
|  |  | Mean IQ |  |  |  | k<4 |
|  |  | AXIS score | 4 | -1.6644 (-16.1882 to 12.8595) | 0.6708 |  |
| **Night waking** | **Social communication problem** | Mean age of participants |  |  |  | k<4 |
|  |  | Percentage of boys |  |  |  | k<4 |
|  |  | Mean IQ |  |  |  | k<4 |
|  |  | AXIS score |  |  |  | k<4 |
|  | **Restricted and repetitive behavior** | Mean age of participants |  |  |  | k<4 |
|  |  | Percentage of boys |  |  |  | k<4 |
|  |  | Mean IQ |  |  |  | k<4 |
|  |  | AXIS score |  |  |  | k<4 |
|  | **Total core symptoms** | Mean age of participants |  |  |  | k<4 |
|  |  | Percentage of boys |  |  |  | k<4 |
|  |  | Mean IQ |  |  |  | k<4 |
|  |  | AXIS score |  |  |  | k<4 |
| **Parasomnias** | **Social communication problem** | Mean age of participants | 4 | -0.0018 (-0.1005 to 0.0970) | 0.9458 |  |
|  |  | Percentage of boys | 4 | 0.0235 (-0.0344 to 0.0813) | 0.2231 |  |
|  |  | Mean IQ |  |  |  | k<4 |
|  |  | AXIS score | 4 | 0.2963 (-9.8374 to 10.4299) | 0.9114 |  |
|  | **Restricted and repetitive behavior** | Mean age of participants |  |  |  | k<4 |
|  |  | Percentage of boys |  |  |  | k<4 |
|  |  | Mean IQ |  |  |  | k<4 |
|  |  | AXIS score |  |  |  | k<4 |
|  | **Total core symptoms** | Mean age of participants |  |  |  | k<4 |
|  |  | Percentage of boys |  |  |  | k<4 |
|  |  | Mean IQ |  |  |  | k<4 |
|  |  | AXIS score |  |  |  | k<4 |
| **Sleep anxiety** | **Social communication problem** | Mean age of participants |  |  |  | k<4 |
|  |  | Percentage of boys |  |  |  | k<4 |
|  |  | Mean IQ |  |  |  | k<4 |
|  |  | AXIS score |  |  |  | k<4 |
|  | **Restricted and repetitive behavior** | Mean age of participants |  |  |  | k<4 |
|  |  | Percentage of boys |  |  |  | k<4 |
|  |  | Mean IQ |  |  |  | k<4 |
|  |  | AXIS score |  |  |  | k<4 |
|  | **Total core symptoms** | Mean age of participants |  |  |  | k<4 |
|  |  | Percentage of boys |  |  |  | k<4 |
|  |  | Mean IQ |  |  |  | k<4 |
|  |  | AXIS score |  |  |  | k<4 |
| **Sleep duration** | **Social communication problem** | Mean age of participants |  |  |  | k<4 |
|  |  | Percentage of boys |  |  |  | k<4 |
|  |  | Mean IQ |  |  |  | k<4 |
|  |  | AXIS score | 4 | 5.8766 (-12.8291 to 24.5824) | 0.309 |  |
|  | **Restricted and repetitive behavior** | Mean age of participants |  |  |  | k<4 |
|  |  | Percentage of boys |  |  |  | k<4 |
|  |  | Mean IQ |  |  |  | k<4 |
|  |  | AXIS score |  |  |  | k<4 |
|  | **Total core symptoms** | Mean age of participants |  |  |  | k<4 |
|  |  | Percentage of boys |  |  |  | k<4 |
|  |  | Mean IQ |  |  |  | k<4 |
|  |  | AXIS score |  |  |  | k<4 |
| **Sleep disordered breathing** | **Social communication problem** | Mean age of participants | 4 | -0.0087 (-0.0286 to 0.0113) | 0.2024 |  |
|  |  | Percentage of boys | 4 | 0.0069 (-0.0262 to 0.0400) | 0.0464 |  |
|  |  | Mean IQ |  |  |  | k<4 |
|  |  | AXIS score | 4 | 0.3646 (-5.1197 to 5.8490) | 0.8017 |  |
|  | **Restricted and repetitive behavior** | Mean age of participants |  |  |  | k<4 |
|  |  | Percentage of boys |  |  |  | k<4 |
|  |  | Mean IQ |  |  |  | k<4 |
|  |  | AXIS score |  |  |  | k<4 |
|  | **Total core symptoms** | Mean age of participants |  |  |  | k<4 |
|  |  | Percentage of boys |  |  |  | k<4 |
|  |  | Mean IQ |  |  |  | k<4 |
|  |  | AXIS score |  |  |  | k<4 |
| **Sleep onset delay** | **Social communication problem** | Mean age of participants |  |  |  | k<4 |
|  |  | Percentage of boys |  |  |  | k<4 |
|  |  | Mean IQ |  |  |  | k<4 |
|  |  | AXIS score |  |  |  | k<4 |
|  | **Restricted and repetitive behavior** | Mean age of participants |  |  |  | k<4 |
|  |  | Percentage of boys |  |  |  | k<4 |
|  |  | Mean IQ |  |  |  | k<4 |
|  |  | AXIS score |  |  |  | k<4 |
|  | **Total core symptoms** | Mean age of participants |  |  |  | k<4 |
|  |  | Percentage of boys |  |  |  | k<4 |
|  |  | Mean IQ |  |  |  | k<4 |
|  |  | AXIS score |  |  |  | k<4 |
| **Total sleep problem** | **Social communication problem** | Mean age of participants | 5 | 0.0553 (-0.0415 to 0.1522) | 0.1667 |  |
|  |  | Percentage of boys | 5 | 0.0449 (-0.0526 to 0.1424) | 0.2392 |  |
|  |  | Mean IQ |  |  |  | k<4 |
|  |  | AXIS score | 6 | -2.1267 (-11.9424 to 7.6889) | 0.5799 |  |
|  | **Restricted and repetitive behavior** | Mean age of participants | 7 | 0.0326 (-0.1058 to 0.1709) | 0.5716 |  |
|  |  | Percentage of boys | 7 | -0.0457 (-0.1423 to 0.0509) | 0.2785 |  |
|  |  | Mean IQ |  |  |  | k<4 |
|  |  | AXIS score | 8 | -5.8259 (-15.8607 to 4.2089) | 0.2052 |  |
|  | **Total core symptoms** | Mean age of participants |  |  |  | k<4 |
|  |  | Percentage of boys |  |  |  | k<4 |
|  |  | Mean IQ |  |  |  | k<4 |
|  |  | AXIS score | 4 | 3.6402 (-24.1721 to 31.4524) | 0.63 |  |

Abbreviations: AXIS= the Appraisal tool for Cross-Sectional Studies, CI=confidence interval, IQ=intelligence quotient, NA=not available; k=the number of studies.

## **eTable 11.2. Statistical results of meta-regression – sleep problems & behavioral problems**

| **Sleep parameters** | **Behavioral parameters** | **Meta-regression moderators** | **k** | **Coefficient (95% CI)** | **p value** | **NA reason** |
| --- | --- | --- | --- | --- | --- | --- |
| **Bedtime resistance** | **Affective/Anxiety problem** |  |  |  |  | k<4 |
|  | **Aggressive/Delinquent problem** | Mean age of participants | 4 | 0.0072 (-0.0351 to 0.0495) | 0.5386 |  |
|  |  | Percentage of boys | 4 | -0.0123 (-0.0521 to 0.0276) | 0.3168 |  |
|  |  | Mean IQ |  |  |  | k<4 |
|  |  | AXIS score |  |  |  | AXIS score of all studies were same |
|  | **Attention problem** | Mean age of participants | 4 | 0.0087 (-0.1390 to 01564) | 0.8235 |  |
|  |  | Percentage of boys | 4 | -0.0406 (-0.1933 to 0.1120) | 0.3708 |  |
|  |  | Mean IQ |  |  |  | k<4 |
|  |  | AXIS score |  |  |  | AXIS score of all studies were same |
|  | **Total behavioral problem** |  |  |  |  | k<4 |
| **Daytime sleepiness** | **Affective/Anxiety problem** | Mean age of participants | 5 | -0.0136 (-0.0414 to 0.141) | 0.2149 |  |
|  |  | Percentage of boys | 5 | -0.0210 (-0.0477 to 0.0057) | 0.0874 |  |
|  |  | Mean IQ |  |  |  | k<4 |
|  |  | AXIS score | 5 | 0.8239 (-6.9833 to 8.6311) | 0.7591 |  |
|  | **Aggressive/Delinquent problem** | Mean age of participants | 5 | 0.0160 (-0.0286 to 0.0607) | 0.3363 |  |
|  |  | Percentage of boys | 5 | 0.0338 (-0.0044 to 0.0721) | 0.0671 |  |
|  |  | Mean IQ |  |  |  | k<4 |
|  |  | AXIS score | 5 | 0.4419 (-8.6762 to 9.5599) | 0.8872 |  |
|  | **Attention problem** | Mean age of participants | 6 | 0.0052 (-0.0285 to 0.0388) | 0.6927 |  |
|  |  | Percentage of boys | 6 | -0.0057 (-0.0336 to 0.0223) | 0.6039 |  |
|  |  | Mean IQ |  |  |  | k<4 |
|  |  | AXIS score | 6 | -1.0359 (-5.6274 to 3.5556) | 0.565 |  |
|  | **Somatic complaints** |  |  |  |  | k<4 |
|  | **Thought problems** |  |  |  |  | k<4 |
|  | **Internalizing problems** |  |  |  |  | k<4 |
|  | **Externalizing problems** |  |  |  |  | k<4 |
|  | **Total behavioral problem** | Mean age of participants | 5 | 0.0122 (-0.0543 to 0.0787) | 0.6006 |  |
|  |  | Percentage of boys | 5 | -0.0110 (-0.1190 to 0.0970) | 0.7664 |  |
|  |  | Mean IQ |  |  |  | k<4 |
|  |  | AXIS score | 5 | -0.8097 (-6.2811 to 4.6618) | 0.6698 |  |
| **Night waking** | **Affective/Anxiety problem** |  |  |  |  | k<4 |
|  | **Aggressive/Delinquent problem** | Mean age of participants | 4 | 0.0162 (-0.0825 to 0.1150) | 0.5523 |  |
|  |  | Percentage of boys | 4 | 0.0468 (0.0269 to 0.0668) | **0.0096** |  |
|  |  | Mean IQ |  |  |  | k<4 |
|  |  | AXIS score |  |  |  | AXIS score of all studies were same |
|  | **Attention problem** | Mean age of participants | 4 | 0.0225 (-0.0471 to 0.0921) | 0.2988 |  |
|  |  | Percentage of boys | 4 | 0.0322 (-0.0246 to 0.0891) | 0.1348 |  |
|  |  | Mean IQ |  |  |  | k<4 |
|  |  | AXIS score |  |  |  | AXIS score of all studies were same |
|  | **Total behavioral problem** |  |  |  |  | k<4 |
| **Parasomnias** | **Affective/Anxiety problem** |  |  |  |  | k<4 |
|  | **Aggressive/Delinquent problem** | Mean age of participants | 4 | 0.0284 (-0.1237 to 0.1806) | 0.5059 |  |
|  |  | Percentage of boys | 4 | 0.0697 (-0.0208 to 0.1581) | 0.0807 |  |
|  |  | Mean IQ |  |  |  | k<4 |
|  |  | AXIS score | 4 | 1.7019 (-14.2626 to 17.7664) |  |  |
|  | **Attention problem** | Mean age of participants | 5 | 0.0040 (-0.0227 to 0.0308) | 0.6628 |  |
|  |  | Percentage of boys | 5 | 0.0101 (-0.0027 to 0.0229) | 0.0868 |  |
|  |  | Mean IQ |  |  |  | k<4 |
|  |  | AXIS score | 5 | 1.2901 (-0.9878 to 3.5680) | 0.1693 |  |
|  | **Somatic complaints** |  |  |  |  | k<4 |
|  | **Thought problems** |  |  |  |  | k<4 |
|  | **Internalizing problems** |  |  |  |  | k<4 |
|  | **Externalizing problems** |  |  |  |  | k<4 |
|  | **Total behavioral problem** | Mean age of participants | 4 | 0.0009 (-0.0646 to 0.0664) | 0.9591 |  |
|  |  | Percentage of boys | 4 | 0.0057 (-0.1333 to 0.1448) | 0.8754 |  |
|  |  | Mean IQ |  |  |  | k<4 |
|  |  | AXIS score | 4 | 0.7752 (-10.0484 to 11.5988) | 0.7871 |  |
| **Sleep anxiety** | **Affective/Anxiety problem** |  |  |  |  | k<4 |
|  | **Aggressive/Delinquent problem** |  |  |  |  | k<4 |
|  | **Attention problem** |  |  |  |  | k<4 |
|  | **Total behavioral problem** |  |  |  |  | k<4 |
| **Sleep duration** | **Affective/Anxiety problem** |  |  |  |  | k<4 |
|  | **Aggressive/Delinquent problem** |  |  |  |  | k<4 |
|  | **Attention problem** | Mean age of participants | 4 | 0.0576 (-0.0021 to 0.1173) | 0.0534 |  |
|  |  | Percentage of boys | 4 | 0.0255 (-0.0731 to 0.1241) | 0.3819 |  |
|  |  | Mean IQ |  |  |  | k<4 |
|  |  | AXIS score | 4 | 2.0554 (-20.2476 to 24.3584) | 0.73 |  |
|  | **Total behavioral problem** |  |  |  |  | k<4 |
| **Sleep disordered breathing** | **Affective/Anxiety problem** | Mean age of participants | 5 | 0.0266 (-0.0470 to 0.1002) | 0.3332 |  |
|  |  | Percentage of boys | 5 | -0.0312 (-0.1618 to 0.0993) | 0.5021 |  |
|  |  | Mean IQ |  |  |  | k<4 |
|  |  | AXIS score | 5 | 2.5183 (-8.7308 to 13.7675) | 0.5276 |  |
|  | **Aggressive/Delinquent problem** | Mean age of participants | 6 | 0.0604 (-0.0295 to 0.1503) | 0.1357 |  |
|  |  | Percentage of boys | 6 | 0.0062 (-0.1609 to 0.1733) | 0.9228 |  |
|  |  | Mean IQ |  |  |  | k<4 |
|  |  | AXIS score | 6 | -5.1791 (-17.5262 to 7.1681) | 0.3089 |  |
|  | **Attention problem** | Mean age of participants | 6 | 0.0149 (-0.0158 to 0.0455) | 0.2492 |  |
|  |  | Percentage of boys | 6 | 0.0036 (-0.0453 to 0.0526) | 0.8463 |  |
|  |  | Mean IQ |  |  |  | k<4 |
|  |  | AXIS score | 6 | -3.1666 (-10.7114 to 4.3782) | 0.3087 |  |
|  | **Somatic complaints** |  |  |  |  | k<4 |
|  | **Internalizing problems** |  |  |  |  | k<4 |
|  | **Externalizing problems** |  |  |  |  | k<4 |
|  | **Total behavioral problem** | Mean age of participants | 5 | 0.0414 (-0.0362 to 0.1191) | 0.1882 |  |
|  |  | Percentage of boys | 5 | 0.0194 (-0.1715 to 0.2102) | 0.7678 |  |
|  |  | Mean IQ |  |  |  | k<4 |
|  |  | AXIS score | 5 | -3.6127 (-15.7708 to 8.5455) | 0.4141 |  |
| **Sleep onset delay** | **Affective/Anxiety problem** |  |  |  |  | k<4 |
|  | **Aggressive/Delinquent problem** |  |  |  |  | k<4 |
|  | **Attention problem** |  |  |  |  | k<4 |
|  | **Total behavioral problem** |  |  |  |  | k<4 |
| **Total sleep problems** | **Affective/Anxiety problem** | Mean age of participants | 6 | -0.0232 (-0.0632 to 0.0168) | 0.1825 |  |
|  |  | Percentage of boys | 6 | 0.0011 (-0.0947 to 0.0968) | 0.9771 |  |
|  |  | Mean IQ |  |  |  | k<4 |
|  |  | AXIS score | 6 | 4.2066 (-4.0439 to 12.4570) | 0.2298 |  |
|  | **Aggressive/Delinquent problem** | Mean age of participants | 6 | 0.0282 (-0.0320 to 0.0883) | 0.2638 |  |
|  |  | Percentage of boys | 6 | -0.0006 (-0.1304 to 0.1292) | 0.9906 |  |
|  |  | Mean IQ |  |  |  | k<4 |
|  |  | AXIS score | 6 | -1.3632 (-11.5429 to 8.8164) | 0.7289 |  |
|  | **Attention problem** | Mean age of participants | 8 | 0.0057 (-0.0236 to 0.0350) | 0.6524 |  |
|  |  | Percentage of boys | 8 | 0.0096 (-0.0189 to 0.0381) | 0.4398 |  |
|  |  | Mean IQ |  |  |  | k<4 |
|  |  | AXIS score | 8 | 0.5981 (-1.0961 to 2.2923) | 0.4208 |  |
|  | **Somatic complaints** |  |  |  |  | k<4 |
|  | **Internalizing problems** | Mean age of participants | 4 | 0.0166 (-0.0632 to 0.0964) | 0.4656 |  |
|  |  | Percentage of boys | 4 | -0.0331 (-0.0901 to 0.0240) | 0.13 |  |
|  |  | Mean IQ |  |  |  | k<4 |
|  |  | AXIS score | 4 | 0.4011 (-10.7793 to 11.5814) | 0.8915 |  |
|  | **Externalizing problems** |  |  |  |  | k<4 |
|  | **Total behavioral problem** | Mean age of participants | 5 | 0.0203 (-0.0255 to 0.0661) | 0.2538 |  |
|  |  | Percentage of boys | 5 | -0.0046 (-0.1268 to 0.1176) | 0.9128 |  |
|  |  | Mean IQ |  |  |  | k<4 |
|  |  | AXIS score | 5 | 1.1213 (-8.3768 to 10.6194) | 0.7321 |  |

Abbreviations: AXIS= the Appraisal tool for Cross-Sectional Studies, CI=confidence interval, IQ=intelligence quotient, NA=not available; k=the number of studies.

## **eTable 11.3. Statistical results of meta-regression analyses - ASD core symptoms & behavioral problems**

| **Behavioral parameters** | **Core symptoms** | **Meta-regression moderators** | **k** | **Coefficient (95% CI)** | **p value** | **NA reason** |
| --- | --- | --- | --- | --- | --- | --- |
| **Affective/Anxiety problem** | **Social communication problem** | Mean age of participants | 4 | 0.0477 (-0.1607 to 0.2561) | 0.4287 |  |
|  |  | Percentage of boys | 4 | -0.0092 (-0.0782 to 0.0597) | 0.6224 |  |
|  |  | Mean IQ |  |  |  | k<4 |
|  |  | AXIS score |  |  |  | AXIS score of all studies were same |
|  | **Restricted and repetitive behavior** | Mean age of participants | 6 | 0.0034 (-0.0774 to 0.0843) | 0.9114 |  |
|  |  | Percentage of boys | 6 | -0.0116 (-0.0325 to 0.0092) | 0.1965 |  |
|  |  | Mean IQ | 4 | -0.0024 (-0.0305 to 0.0257) | 0.7487 |  |
|  |  | AXIS score |  |  |  | AXIS score of all studies were same |
|  | **Total core symptoms** | Mean age of participants |  |  |  | k<4 |
|  |  | Percentage of boys |  |  |  | k<4 |
|  |  | Mean IQ |  |  |  | k<4 |
|  |  | AXIS score |  |  |  | k<4 |
| **Aggressive/Delinquent problem** | **Social communication problem** | Mean age of participants |  |  |  | k<4 |
|  |  | Percentage of boys |  |  |  | k<4 |
|  |  | Mean IQ |  |  |  | k<4 |
|  |  | AXIS score |  |  |  | k<4 |
|  | **Restricted and repetitive behavior** | Mean age of participants | 5 | 0.0680 (-0.1448 to 0.2808) | 0.3841 |  |
|  |  | Percentage of boys | 5 | -0.0102 (-0.0575 to 0.0371) | 0.5417 |  |
|  |  | Mean IQ |  |  |  | k<4 |
|  |  | AXIS score | 5 | -6.9940 (-19.5374 to 5.5495) | 0.1741 |  |
|  | **Total core symptoms** | Mean age of participants |  |  |  | k<4 |
|  |  | Percentage of boys |  |  |  | k<4 |
|  |  | Mean IQ |  |  |  | k<4 |
|  |  | AXIS score |  |  |  | k<4 |
| **Attention problem** | **Social communication problem** | Mean age of participants | 4 | 0.0131 (-0.1673 to 0.1935) | 0.785 |  |
|  |  | Percentage of boys | 4 | -0.0132 (-0.0503 to 0.0239) | 0.2658 |  |
|  |  | Mean IQ |  |  |  | k<4 |
|  |  | AXIS score |  |  |  | AXIS score of all studies were same |
|  | **Restricted and repetitive behavior** | Mean age of participants | 6 | 0.0034 (-0.0774 to 0.0843) | 0.9114 |  |
|  |  | Percentage of boys | 6 | -0.0116 (-0.0325 to 0.0092) | 0.1965 |  |
|  |  | Mean IQ | 4 | -0.0024 (-0.0305 to 0.0257) | 0.7487 |  |
|  |  | AXIS score |  |  |  | AXIS score of all studies were same |
| **Somatic complaints** | **Total core symptoms** | Mean age of participants |  |  |  | k<4 |
|  |  | Percentage of boys |  |  |  | k<4 |
|  |  | Mean IQ |  |  |  | k<4 |
|  |  | AXIS score |  |  |  | k<4 |
| **Total behavioral problem** | **Social communication problem** | Mean age of participants |  |  |  | k<4 |
|  |  | Percentage of boys |  |  |  | k<4 |
|  |  | Mean IQ |  |  |  | k<4 |
|  |  | AXIS score |  |  |  | k<4 |
|  | **Restricted and repetitive behavior** | Mean age of participants |  |  |  | k<4 |
|  |  | Percentage of boys |  |  |  | k<4 |
|  |  | Mean IQ |  |  |  | k<4 |
|  |  | AXIS score |  |  |  | k<4 |
|  | **Total core symptoms** | Mean age of participants |  |  |  | k<4 |
|  |  | Percentage of boys |  |  |  | k<4 |
|  |  | Mean IQ |  |  |  | k<4 |
|  |  | AXIS score |  |  |  | k<4 |

Abbreviations: AXIS= the Appraisal tool for Cross-Sectional Studies, CI=confidence interval, IQ=intelligence quotient, NA=not available; k=the number of studies.

# **eAppendix 12. Statistical results of the subgroup analyses - Medication use status (note that subgroup analysis for correlations between ASD core symptoms & behavioral problems was unavailable)**

## **eTable 12.1. Statistical results of the subgroup analyses – sleep problems & ASD core symptoms**

| **Sleep parameters** | **Core symptoms** | **Medication Use** | **k** | **Meta-analysis** | **Heterogeneity** | | **P**  **(test for subgroup differences)** | **NA reason** |
| --- | --- | --- | --- | --- | --- | --- | --- | --- |
|  |  |  |  | **Correlation (95% CI)** | **Q value** | ***I^2^* (%)** |  |  |
| **Bedtime resistance** | **Social and communication problem** | Yes | 2 | 0.0022 (-0.5516 to 0.5546) | 0 | 0 | 0.4251 |  |
|  |  | No | 1 | 0.0660 (-0.0580 to 0.1880) |  |  |  |  |
|  | **Restricted and repetitive behavior** | Yes | 1 | 0.4330 (0.3269 to 0.5283) |  |  | <0.0001 |  |
|  |  | No | 1 | 0.0600 (-0.0574 to 0.1758) |  |  |  |  |
|  | **Total core symptoms** | Yes | 1 | 0.0000 (-0.1170 to 0.1170) |  |  | 0.0009 |  |
|  |  | No | 2 | 0.1966 (0.1849 to 0.2083) | 0 | 0 |  |  |
| **Daytime sleepiness** | **Social and communication problem** | Yes | 4 | 0.0581 (-0.1081 to 0.2211) | 3.01 | 0.5 | 0.0427 |  |
|  |  | No | 1 | -0.1080 (-0.2285 to 0.0158) |  |  |  |  |
|  | **Restricted and repetitive behavior** | Yes | 2 | 0.1491 (-0.9792 to 0.9886) | 9.29 | 89.2 | 0.8394 |  |
|  |  | No | 1 | 0.1090 (-0.0148 to 0.2295) |  |  |  |  |
|  | **Total core symptoms** | Yes | 2 | 0.0202 (-0.7684 to 0.7844) | 1.85 | 45.8 | 0.5552 |  |
|  |  | No | 2 | 0.0968 (-0.8326 to 0.8833) | 3.19 | 68.7 |  |  |
| **Night waking** | **Social and communication problem** | Yes | 2 | -0.0281 (-0.3474 to 0.2970) | 0.23 | 0 | 0.0015 |  |
|  |  | No | 1 | 0.1880 (0.0660 to 0.3045) |  |  |  |  |
|  | **Restricted and repetitive behavior** | Yes | 1 | 0.0600 (-0.0574 to 0.1758) |  |  | 0.2137 |  |
|  |  | No | 1 | 0.1670 (0.0443 to 0.2847) |  |  |  |  |
|  | **Total core symptoms** | Yes | 1 | -0.0100 (-0.1269 to 0.1071) |  |  | 0.0002 |  |
|  |  | No | 2 | 0.2641 (-0.2823 to 0.6811) |  |  |  |  |
| **Parasomnias** | **Social and communication problem** | Yes | 3 | -0.0310 (-0.3709 to 0.3162) | 2.1 | 4.6 | 0.4109 |  |
|  |  | No | 1 | 0.0550 (-0.0690 to 0.1774) |  |  |  |  |
|  | **Restricted and repetitive behavior** | Yes | 4 | -0.0309 (-0.2348 to 0.1756) | 2.1 | 0 | 0.3453 |  |
|  |  | No | 1 | 0.0550 (-0.0690 to 0.1774) |  |  |  |  |
|  | **Total core symptoms** | Yes | 1 | -0.0500 (-0.2603 to 0.1649) |  |  | 0.0433 |  |
|  |  | No | 2 | 0.1719 ( 0.0769 to 0.2637) | 0.02 | 0 |  |  |
| **Sleep anxiety** | **Social and communication problem** | Yes | 1 | 0.0600 (-0.1969 to 0.3092) |  |  | 0.7945 |  |
|  |  | No | 1 | 0.0980 (-0.0259 to 0.2189) |  |  |  |  |
|  | **Restricted and repetitive behavior** | Yes |  |  |  |  |  | k<2 |
|  |  | No |  |  |  |  |  |  |
|  | **Total core symptoms** | Yes |  |  |  |  |  | All included studies involved medication-naive patients |
|  |  | No |  |  |  |  |  |  |
| **Sleep duration** | **Social and communication problem** | Yes | 3 | 0.1075 (-0.4747 to 0.6242) | 9.04 | 77.9 | 0.37 |  |
|  |  | No | 1 | -0.0340 (-0.1569 to 0.0900) |  |  |  |  |
|  | **Restricted and repetitive behavior** | Yes | 2 | -0.1702 (-0.8763 to 0.7682) | 1.42 | 29.3 | 0.0322 |  |
|  |  | No | 1 | 0.0700 (-0.0540 to 0.1919) |  |  |  |  |
|  | **Total core symptoms** | Yes | 2 | -0.1678 (-0.6162 to 0.3628) | 0.3 | 0 | <0.0001 |  |
|  |  | No | 1 | 0.2210 ( 0.1002 to 0.3354) |  |  |  |  |
| **Sleep disordered breathing** | **Social and communication problem** | Yes | 3 | -0.0152 (-0.1085 to 0.0783) | 0.33 | 0 | 0.7453 |  |
|  |  | No | 1 | -0.0370 (-0.1598 to 0.0870) |  |  |  |  |
|  | **Restricted and repetitive behavior** | Yes | 1 | -0.2000 (-0.3098 to -0.0850) |  |  | <0.0001 |  |
|  |  | No | 1 | 0.2920 ( 0.1747 to 0.4011) |  |  |  |  |
|  | **Total core symptoms** | Yes | 1 | -0.0900 (-0.2049 to 0.0273) |  |  | 0.3464 |  |
|  |  | No | 2 | 0.0666 (-0.9571 to 0.9669) | 7.36 | 86.4 |  |  |
| **Sleep onset delay** | **Social and communication problem** | Yes | 1 | 0.3600 (0.1167 to 0.5625) |  |  | 0.1403 |  |
|  |  | No | 1 | 0.1590 (0.0361 to 0.2771) |  |  |  |  |
|  | **Restricted and repetitive behavior** | Yes |  |  |  |  |  | k<2 |
|  |  | No |  |  |  |  |  |  |
|  | **Total core symptoms** | Yes |  |  |  |  |  | All included studies involved medication-naive patients |
|  |  | No |  |  |  |  |  |  |
| **Total sleep problem** | **Social and communication problem** | Yes | 4 | 0.0831 (-0.3024 to 0.4452) | 7.03 | 57.3 | 0.5483 |  |
|  |  | No | 2 | 0.2266 (-0.9855 to 0.9942) | 8.83 | 88.7 |  |  |
|  | **Restricted and repetitive behavior** | Yes | 6 | 0.1391 (-0.2963 to 0.5267) | 39.97 | 87.5 | 0.1617 |  |
|  |  | No | 2 | 0.3866 (-0.5531 to 0.8934) | 1.53 | 34.6 |  |  |
|  | **Total core symptoms** | Yes | 2 | -0.0851 (-0.5752 to 0.4499) | 0.33 | 0 | 0.0266 |  |
|  |  | No | 2 | 0.2634 (-0.9333 to 0.9768) | 7.19 | 86.1 |  |  |

Abbreviations: CI=confidence interval, NA=not available, k=the number of studies

## **eTable 12.2. Statistical results of the subgroup analyses – sleep problems & behavioral problems**

| **Sleep parameters** | **Behavioral parameters** | **Medication Use** | **k** | **Meta-analysis** | **Heterogeneity** | | **P**  **(test for subgroup differences)** | **NA reason** |
| --- | --- | --- | --- | --- | --- | --- | --- | --- |
|  |  |  |  | **Correlation (95% CI)** | **Q value** | ***I^2^* (%)** |  |  |
| **Bedtime resistance** | **Affective/Anxiety problem** | Yes | 2 | 0.2383 (-0.1073 to 0.5324) | 0.26 | 0 | 0.3861 |  |
|  |  | No | 1 | 0.1810 (0.0587 to 0.2979) |  |  |  |  |
|  | **Aggressive/Delinquent problem** | Yes | 3 | 0.1626 (0.0615 to 0.2603) | 0.47 | 0 | 0.3868 |  |
|  |  | No | 1 | 0.2190 (0.0981 to 0.3335) |  |  |  |  |
|  | **Attention problem** | Yes | 3 | 0.1754 (-0.3408 to 0.6103) | 6.67 | 70 | 0.6216 |  |
|  |  | No | 1 | 0.2410 (0.1210 to 0.3540) |  |  |  |  |
|  | **Total behavioral problem** | Yes | 1 | 0.3500 (0.1054 to 0.5546) |  |  | 0.2558 |  |
|  |  | No | 1 | 0.1960 (0.0742 to 0.3120) |  |  |  |  |
| **Daytime sleepiness** | **Affective/Anxiety problem** | Yes | 4 | 0.2718 (0.1572 to 0.3791) | 1.59 | 0 | 0.3853 |  |
|  |  | No | 1 | 0.3300 (0.2152 to 0.4358) |  |  |  |  |
|  | **Aggressive/Delinquent problem** | Yes |  |  |  |  |  |  |
|  |  | No |  |  |  |  |  |  |
|  | **Attention problem** | Yes | 5 | 0.1528 (0.0542 to 0.2485) | 2.62 | 0 | 0.3309 |  |
|  |  | No | 1 | 0.0830 (-0.0410 to 0.2045) |  |  |  |  |
|  | **Somatic complaints** | Yes |  |  |  |  |  | k<2 |
|  |  | No |  |  |  |  |  |  |
|  | **Thought problems** | Yes |  |  |  |  |  | k<2 |
|  |  | No |  |  |  |  |  |  |
|  | **Internalizing problems** | Yes |  |  |  |  |  | All included studies involved medication-use patients |
|  |  | No |  |  |  |  |  |  |
|  | **Externalizing problems** | Yes |  |  |  |  |  | All included studies involved medication-use patients |
|  |  | No |  |  |  |  |  |  |
|  | **Total behavioral problem** | Yes | 4 | 0.3575 (0.2778 to 0.4324) | 0.7 | 0 | 0.0004 |  |
|  |  | No | 1 | 0.1280 (0.0045 to 0.2477) |  |  |  |  |
| **Night waking** | **Affective/Anxiety problem** | Yes | 2 | 0.2386 (-0.0121 to 0.4611) | 0.14 | 0 | 0.0848 |  |
|  |  | No | 1 | 0.1280 (0.0045 to 0.2477) |  |  |  |  |
|  | **Aggressive/Delinquent problem** | Yes | 3 | 0.3048 (0.1446 to 0.4494) | 1.28 | 0 | 0.0104 |  |
|  |  | No | 1 | 0.1230 (-0.0006 to 0.2429) |  |  |  |  |
|  | **Attention problem** | Yes | 3 | 0.2509 (0.0449 to 0.4364) | 1.87 | 0 | 0.1059 |  |
|  |  | No | 1 | 0.1260 (0.0025 to 0.2457) |  |  |  |  |
|  | **Total behavioral problem** | Yes | 1 | 0.3000 (0.0499 to 0.5147) |  |  | 0.613 |  |
|  |  | No | 1 | 0.2310 (0.1106 to 0.3447) |  |  |  |  |
| **Parasomnias** | **Affective/Anxiety problem** | Yes | 2 | 0.2197 (-0.7452 to 0.8872) | 0.6 | 0 | 0.7907 |  |
|  |  | No | 1 | 0.1910 (0.0691 to 0.3073) |  |  |  |  |
|  | **Aggressive/Delinquent problem** | Yes | 3 | 0.2865 (-0.1261 to 0.6146) | 2.55 | 21.6 | 0.006 |  |
|  |  | No | 1 | -0.0260 (-0.1491 to 0.0979) |  |  |  |  |
|  | **Attention problem** | Yes | 4 | 0.2478 (0.1415 to 0.3485) | 0.83 | 0 | 0.8508 |  |
|  |  | No | 1 | 0.2350 (0.1148 to 0.3485) |  |  |  |  |
|  | **Somatic complaints** | Yes |  |  |  |  |  | k<2 |
|  |  | No |  |  |  |  |  |  |
|  | **Thought problems** | Yes |  |  |  |  |  | k<2 |
|  |  | No |  |  |  |  |  |  |
|  | **Internalizing problems** | Yes |  |  |  |  |  | All included studies involved medication-use patients |
|  |  | No |  |  |  |  |  |  |
|  | **Externalizing problems** | Yes |  |  |  |  |  | All included studies involved medication-use patients |
|  |  | No |  |  |  |  |  |  |
|  | **Total behavioral problem** | Yes | 3 | 0.2920 (0.2099 to 0.3699) | 0.21 | 0 | 0.088 |  |
|  |  | No | 1 | 0.1850 (0.0629 to 0.3017) |  |  |  |  |
| **Sleep anxiety** | **Affective/Anxiety problem** | Yes | 1 | 0.3400 (0.0942 to 0.5467) |  |  | 0.199 |  |
|  |  | No | 1 | 0.1640 (0.0413 to 0.2819) |  |  |  |  |
|  | **Aggressive/Delinquent problem** | Yes | 2 | 0.3013 (-0.0351 to 0.5764) | 0.1 | 0 | 0.0027 |  |
|  |  | No | 1 | 0.1040 (-0.0198 to 0.2247) |  |  |  |  |
|  | **Attention problem** | Yes | 2 | 0.2024 (-0.9948 to 0.9977) | 8.23 | 87.9 | 0.335 |  |
|  |  | No | 1 | 0.4250 (0.3181 to 0.5212) |  |  |  |  |
|  | **Total behavioral problem** | Yes | 1 | 0.4400 (0.2095 to 0.6242) |  |  | 0.3087 |  |
|  |  | No | 1 | 0.3120 (0.1960 to 0.4194) |  |  |  |  |
| **Sleep duration** | **Affective/Anxiety problem** | Yes | 1 | 0.1700 (-0.0877 to 0.4064) |  |  | 0.7072 |  |
|  |  | No | 1 | 0.2230 (0.1022 to 0.3373) |  |  |  |  |
|  | **Aggressive/Delinquent problem** | Yes | 2 | 0.3413 (-0.0007 to 0.6119) | 0.11 | 0 | 0.0099 |  |
|  |  | No | 1 | 0.1750 ( 0.0526 to 0.2923) |  |  |  |  |
|  | **Attention problem** | Yes | 3 | 0.2152 (-0.1437 to 0.5241) | 3.1 | 35.4 | 0.0081 |  |
|  |  | No | 1 | -0.0610 (-0.1832 to 0.0630) |  |  |  |  |
|  | **Total behavioral problem** | Yes | 1 | 0.4200 (0.1859 to 0.6090) |  |  | 0.0288 |  |
|  |  | No | 1 | 0.1260 (0.0025 to 0.2457) |  |  |  |  |
| **Sleep disordered breathing** | **Affective/Anxiety problem** | Yes | 3 | 0.1616 (-0.0151 to 0.3286) | 1.19 | 0 | 0.7857 |  |
|  |  | No | 2 | 0.2253 (-0.9929 to 0.9971) | 11.3 | 91.1 |  |  |
|  | **Aggressive/Delinquent problem** | Yes | 4 | 0.1193 (-0.1020 to 0.3293) | 5.78 | 48.1 | 0.7381 |  |
|  |  | No | 2 | 0.2575 (-0.9999 to 1.0000) | 35.23 | 97.2 |  |  |
|  | **Attention problem** | Yes | 4 | 0.2099 (0.1166 to 0.2995) | 1.16 | 0 | 0.8751 |  |
|  |  | No | 2 | 0.2295 (-0.8826 to 0.9522) | 3.3 | 69.7 |  |  |
|  | **Somatic complaints** | Yes |  |  |  |  |  | k<2 |
|  |  | No |  |  |  |  |  |  |
|  | **Internalizing problems** | Yes | 2 | 0.1294 (0.0993 to 0.1593) | 0 | 0 | 0.0053 |  |
|  |  | No | 1 | 0.4520 (0.2320 to 0.6280) |  |  |  |  |
|  | **Externalizing problems** | Yes | 2 | 0.3277 (-0.9936 to 0.9984) | 3.13 | 68 | 0.4952 |  |
|  |  | No | 1 | 0.4880 ( 0.2752 to 0.6552) |  |  |  |  |
|  | **Total behavioral problem** | Yes | 3 | 0.2259 (-0.0295 to 0.4536) | 1.88 | 0 | 0.7938 |  |
|  |  | No | 2 | 0.2964 (-0.9972 to 0.9992) | 15.75 | 93.6 |  |  |
| **Sleep onset delay** | **Affective/Anxiety problem** | Yes | 1 | 0.0300 (-0.2256 to 0.2818) |  |  | 0.1436 |  |
|  |  | No | 1 | 0.2400 ( 0.1200 to 0.3531) |  |  |  |  |
|  | **Aggressive/Delinquent problem** | Yes | 2 | 0.1168 (-0.5261 to 0.6747) | 0.41 | 0 | 0.2016 |  |
|  |  | No | 1 | 0.2210 ( 0.1002 to 0.3354) |  |  |  |  |
|  | **Attention problem** | Yes | 2 | 0.1709 (-0.8137 to 0.9020) | 1.42 | 29.5 | 0.7964 |  |
|  |  | No | 1 | 0.2010 ( 0.0794 to 0.3167) |  |  |  |  |
|  | **Total behavioral problem** | Yes | 1 | 0.2900 (0.0389 to 0.5066) |  |  | 0.6488 |  |
|  |  | No | 1 | 0.3500 (0.2367 to 0.4539) |  |  |  |  |
| **Total sleep problem** | **Affective/Anxiety problem** | Yes | 4 | 0.4479 ( 0.1765 to 0.6560) | 7.77 | 6.14 | 0.7824 |  |
|  |  | No | 2 | 0.4247 (-0.0660 to 0.7500) | 0.52 | 0 |  |  |
|  | **Aggressive/Delinquent problem** | Yes | 4 | 0.3970 ( 0.3360 to 0.4548) | 0.74 | 0 | 0.8767 |  |
|  |  | No | 2 | 0.4370 (-0.9981 to 0.9997) | 18.94 | 94.7 |  |  |
|  | **Attention problem** | Yes | 6 | 0.3594 ( 0.2698 to 0.4428) | 4.86 | 0 | 0.1351 |  |
|  |  | No | 2 | 0.2714 (-0.3732 to 0.7393) | 0.86 | 0 |  |  |
|  | **Somatic complaints** | Yes |  |  |  |  |  | All included studies involved medication-use patients |
|  |  | No |  |  |  |  |  |  |
|  | **Internalizing problems** | Yes | 3 | 0.3557 (0.0326 to 0.6114) | 2.73 | 26.7 | 0.4384 |  |
|  |  | No | 1 | 0.4530 (0.2332 to 0.6288) |  |  |  |  |
|  | **Externalizing problems** | Yes | 2 | 0.3259 (0.1074 to 0.5144) | 0.07 | 0 | 0.0329 |  |
|  |  | No | 1 | 0.5470 (0.3480 to 0.6988) |  |  |  |  |
|  | **Total behavioral problem** | Yes | 3 | 0.4516 ( 0.3299 to 0.5585) | 0.58 | 0 | 0.8532 |  |
|  |  | No | 2 | 0.4296 (-0.8763 to 0.9792) | 4.11 | 75.6 |  |  |

Abbreviations: CI=confidence interval, NA=not available to k=the number of studies.
